# Metabolic excretion associated with nutrient–growth dysregulation promotes the rapid evolution of an overt metabolic defect

**DOI:** 10.1371/journal.pbio.3000757

**Published:** 2020-08-24

**Authors:** Robin Green, Sonal, Lin Wang, Samuel F. M. Hart, Wenyun Lu, David Skelding, Justin C. Burton, Hanbing Mi, Aric Capel, Hung Alex Chen, Aaron Lin, Arvind R. Subramaniam, Joshua D. Rabinowitz, Wenying Shou

**Affiliations:** 1 Division of Basic Sciences, Fred Hutchinson Cancer Research Center, Seattle, Washington, United States of America; 2 Department of Chemistry and Lewis-Sigler Institute for Integrative Genomics, Princeton University, Princeton, New Jersey, United States of America; Wageningen University, NETHERLANDS

## Abstract

In eukaryotes, conserved mechanisms ensure that cell growth is coordinated with nutrient availability. Overactive growth during nutrient limitation (“nutrient–growth dysregulation”) can lead to rapid cell death. Here, we demonstrate that cells can adapt to nutrient–growth dysregulation by evolving major metabolic defects. Specifically, when yeast lysine-auxotrophic mutant *lys*^−^ encountered lysine limitation, an evolutionarily novel stress, cells suffered nutrient–growth dysregulation. A subpopulation repeatedly evolved to lose the ability to synthesize organosulfurs (*lys*^−^*orgS*^−^). Organosulfurs, mainly reduced glutathione (GSH) and GSH conjugates, were released by *lys*^−^ cells during lysine limitation when growth was dysregulated, but not during glucose limitation when growth was regulated. Limiting organosulfurs conferred a frequency-dependent fitness advantage to *lys*^−^*orgS*^−^ by eliciting a proper slow growth program, including autophagy. Thus, nutrient–growth dysregulation is associated with rapid organosulfur release, which enables the selection of organosulfur auxotrophy to better tune cell growth to the metabolic environment. We speculate that evolutionarily novel stresses can trigger atypical release of certain metabolites, setting the stage for the evolution of new ecological interactions.

## Abstract 2

In eukaryotes, conserved mechanisms ensure that cell growth is tuned to nutrient availability. This study shows that when cells cannot do this, novel metabolic interactions can rapidly evolve, and metabolites released from some cells can open a new route for other cells to evolve advantageous metabolic defects that better tune growth.

## Introduction

All organisms must coordinate growth with the availability of nutrients. In eukaryotes, when nutrients are abundant, cells express growth-promoting genes and grow. When nutrients are limited, cells halt growth and instead launch stress-response programs to survive. Mechanisms that ensure this coordination between nutrient availability and growth are conserved across eukaryotes [1].

**Fig 1:**
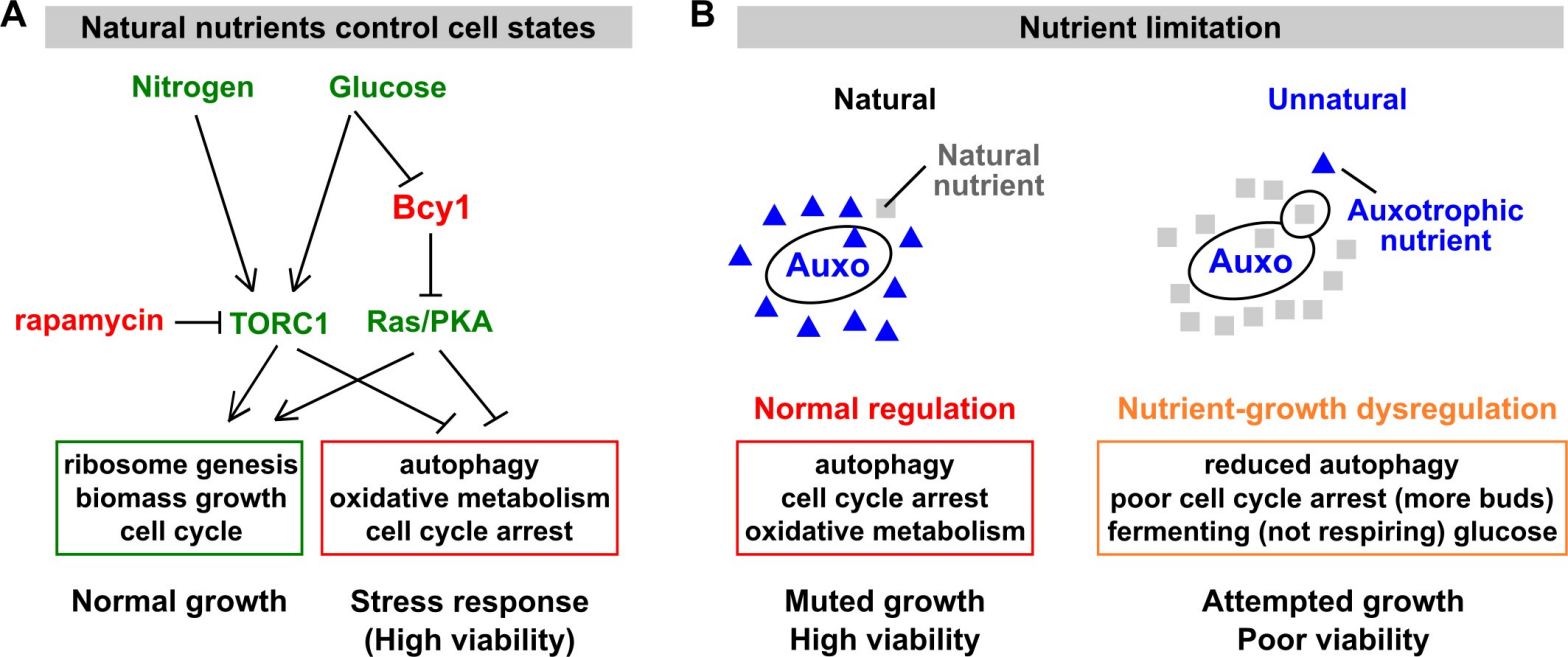
Nutrient–growth regulation and dysregulation. (A) Natural nutrients control the growth or the stress-response state of a cell (reviewed in [2]). Growth stimulatory molecules are colored green, and growth inhibitory molecules are colored red. Broadly speaking, the presence of natural essential nutrients (e.g., nitrogen, glucose, sulfur, phosphorus) activates the TORC1 pathway. Glucose additionally activates the Ras/PKA pathway, although this activation is transient if essential natural nutrients are incomplete [10]. For simplicity, we have omitted other input pathways (e.g., in [11]). TORC1 and Ras/PKA pathways activate cell growth (green box) and inhibit stress response (red box). Conversely, the shortage of a natural nutrient or inhibition of TORC1 (via rapamycin) triggers stress responses, including cell-cycle arrest, autophagy, and oxidative metabolism. Note that the mRNA levels of greater than a quarter of yeast genes are linearly correlated with growth rate, independent of the nature of the nutrient limitation [4,12]. During slow growth, repressed genes include those involved in ribosome synthesis, translation initiation, and protein and RNA metabolism, whereas induced genes are involved in autophagy, lipid metabolism, and oxidative metabolism (including those annotated to peroxisomes and the peroxisomal matrix) [4,9,13,14]. (B) Left: when limited for a natural nutrient, an auxotroph responds properly with muted growth (red box) and survives with high viability. Right: when limited for the auxotrophic nutrient, an auxotroph suffers nutrient–growth dysregulation (orange box). Despite nutrient limitation, cells experience poor cell-cycle arrest, suffer reduced autophagy, and metabolize glucose via fermentation instead of respiration. Consequently, these cells suffer low viability. S1 Text offers additional discussions. Auxo, auxotroph; Bcy1, bypass of cyclic-AMP requirement protein 1; PKA, protein kinase A; TORC1, target of rapamycin complex 1.

The budding yeast *Saccharomyces cerevisiae* senses and responds to the availability of natural nutrients—nutrients that must be supplied from the environment [2]. Examples of natural nutrients include carbon, nitrogen, phosphorus, and sulfur. When natural nutrients are abundant, the TORC1 (target of rapamycin complex 1) pathway is activated. If the carbon source happens to be glucose, the Ras/protein kinase A (PKA) pathway is additionally activated [2,3]. Activated TORC1 and PKA pathways promote growth-related processes, including ribosome synthesis, biomass accumulation, and cell division (Fig 1A, green box). Simultaneously, TORC1 and PKA inhibit stress-response processes (Fig 1A, red box). Thus, abundant natural nutrients set the cell state to the growth mode. In contrast, when one of the essential nutrients is missing, the cell state is switched to the stress-response mode (Fig 1A and 1B, red box): cells up-regulate stress-responsive genes and acquire enhanced resistance to heat and to high osmolarity. Cell division is arrested in an unbudded state; oxidative metabolism is elevated, wherein cells consume more oxygen and do not ferment glucose into ethanol [4–7]. Additionally, cells engage in autophagy, a stress survival process involving degradation and recycling of cytosol and organelles [5,8,9]. Thus, proper nutrient–growth regulation allows cells to grow when natural nutrients are abundant and to maintain high viability when natural nutrients are scarce or lacking.

In contrast, “unnatural limitation” occurs when a yeast auxotrophic mutant is limited for the metabolite it can no longer make (e.g., a *leu*^−^ mutant limited for leucine). In general, auxotrophic limitations are unnatural. This is because in the wild, yeast strains are diploid and thus remain prototrophic (capable of synthesizing a metabolite) even if one of the 2 gene copies for metabolite synthesis is impaired. In special cases, auxotrophic limitation might mimic natural limitation; e.g., intracellular glutamine level may reflect nitrogen availability [15], whereas intracellular methionine level may reflect sulfur or general amino acid availability [16,17]. Leucine, whose biosynthesis occurs in both cytoplasm and mitochondria, may monitor the tricarboxylic acid (TCA) cycle and the ADP/ATP ratio [18]. Thus, some of the mechanisms for sensing and responding to these amino acids [19,20] might actually function to sense natural nutrients.

Nutrient–growth regulation can become dysfunctional during unnatural auxotrophic limitation. During unnatural limitation, cells fail to arrest the cell cycle, metabolize glucose through fermentation instead of respiration, and suppress the transcription of stress-response genes such as vacuolar genes, autophagy genes, as well as respiration-related genes involved in mitochondria and the TCA cycle [4,6,7,21] (Fig 1B, orange box). Cells engage in significantly less autophagic activity under unnatural auxotrophic starvation compared to natural nitrogen starvation (Table II in [22]). Unnaturally starved cells suffer poor viability, which can be rescued by inhibiting growth [6,23,24], a phenotype we define as “nutrient–growth dysregulation.”

Multiple experiments support this model of nutrient–growth regulation and dysregulation (Fig 1B, left and right panels, respectively). Inactivating the TORC1 pathway, either by using its inhibitor rapamycin or by deleting *TOR1* or its downstream effector *SCH9* (encoding a serine/threonine protein kinase), allowed auxotrophic cells to better survive unnatural limitations [23,24]. Deleting *PPM1* (protein phosphatase methyltransferase gene 1), a gene that promotes growth and inhibits autophagy, helped *leu*^−^ cells to survive unnatural leucine starvation, but not natural phosphate starvation [23,24]. Deletion of *BCY1* (bypass of cyclic-AMP requirement gene 1), which resulted in constitutively active PKA, caused failed cell-cycle arrest and poor viability during nitrogen starvation [25,26]. Finally, auxotrophic cells under unnatural limitation died much faster when the carbon source was glucose (which additionally activates PKA) than when the carbon source was poor, such as ethanol or glycerol [23].

Because nutrient–growth regulation is conserved across eukaryotes [1], here we investigate how cells suffering nutrient–growth dysregulation might evolve, taking advantage of the *lys2Δ* strain (“*lys*^−^”) of *S*. *cerevisiae*.

## Results

### *lys*^−^ cells under lysine limitation suffer nutrient–growth dysregulation

Previous work has established that *lys*^−^ cells limited for lysine displayed hallmarks of nutrient–growth dysregulation [21]. Unlike prototrophic cells limited for a natural nutrient, lysine-limited *lys*^−^ cells suffered decoupling between the cell division cycle and biomass production, fermented glucose in abundant oxygen, and suppressed the transcription of autophagy genes and mitochondrial and respiration genes [21]. Note that oxidative metabolism during nutrient limitation is important for cell viability since deleting genes involved in respiration and mitochondrion organization rendered cells sensitive to glucose starvation [5].

**Fig 2:**
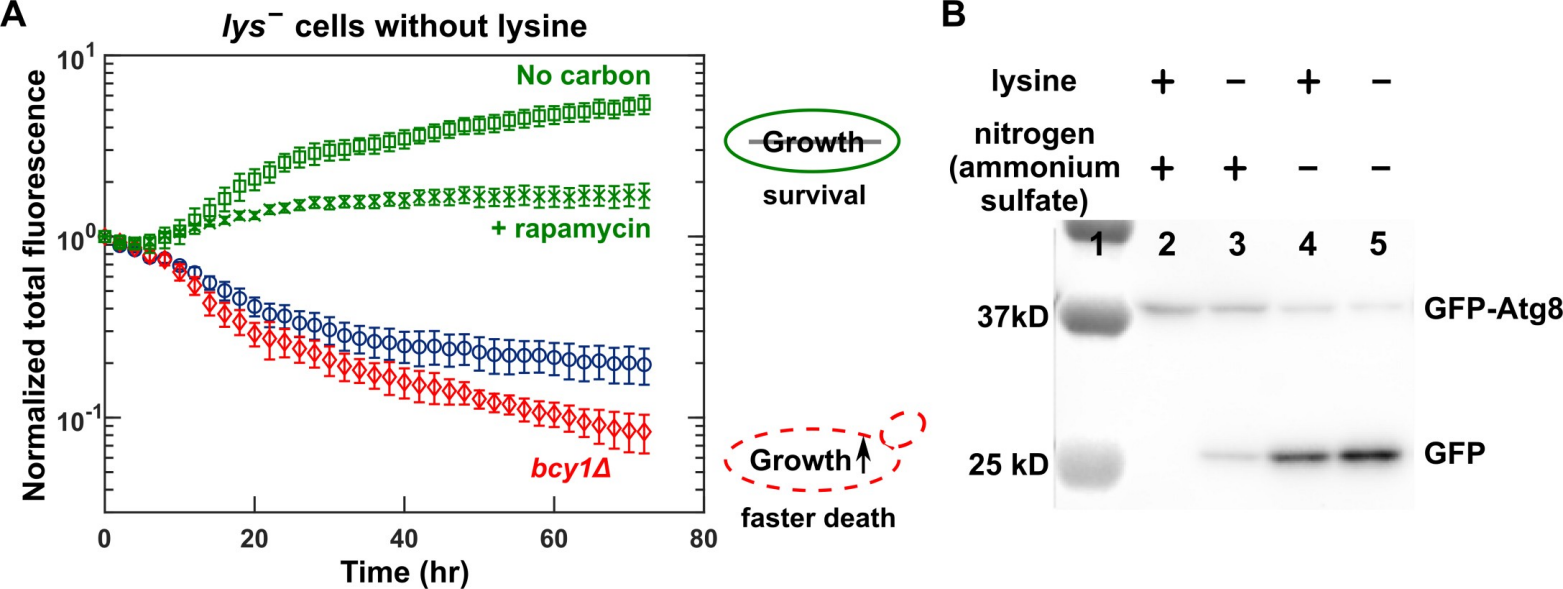
*lys*^−^ cells suffer nutrient–growth dysregulation when limited for lysine. (A) The poor viability of *lys*^−^ cells during lysine starvation is rescued by inhibiting growth and is exacerbated by activating growth. Exponentially growing mCherry-expressing *lys*^−^ (WY2490) cells were washed and starved for either only lysine or both lysine and glucose for 3 hours to deplete cellular storage and cultured and imaged in indicated environments (Methods, “Fluorescence microscopy”). Total fluorescence (normalized to the initial value) approximates total biomass [27]. In the absence of lysine, *lys*^−^ cells died rapidly when glucose was abundant (2%, blue circles). This rapid death could be rescued if we inhibited growth by adding the TORC1 inhibitor rapamycin (1 μM, green crosses) or by simultaneously starving for glucose (“No carbon,” green squares). Rapid death was exacerbated if we activated growth by deleting the PKA inhibitor *BCY1* (red diamonds). Error bars correspond to 2 standard deviations for 6 replicate wells. Plotted data are provided as S1 Data. (B) *lys*^−^ cells engage in less autophagy during lysine starvation compared with during nitrogen starvation. Lane 1 is the protein ladder. *lys*^−^ cells expressing GFP-Atg8 (WY2521) were grown to exponential phase (Lane 2) and washed free of nutrients. Cells were then starved for only lysine (Lane 3), only nitrogen (Lane 4), or both lysine and nitrogen (Lane 5) for 8 hours. Cell extracts were subjected to western blotting using anti-GFP antibodies (Methods, “Autophagy assay”). High GFP/GFP-Atg8 ratio indicates high autophagy activity. Data are representative of 3 trials. Atg8, autophagy-related protein 8; *bcy1Δ*, a mutant with deletion of bypass of cyclic-AMP requirement gene 1; GFP, green fluorescent protein; PKA, protein kinase A; TORC1, target of rapamycin complex 1.

Consistent with nutrient–growth dysregulation, lysine-limited *lys*^−^ cells suffer low viability that can be rescued by inhibiting cell growth. Specifically, we used a lysine-auxotrophic *S*. *cerevisiae* strain *lys*^−^, engineered to harbor a *lys2* deletion mutation and also to express the fluorescent protein mCherry (WY1335/WY2490, S1 Table). When starved for lysine, *lys*^−^ cells rapidly lost fluorescence (Fig 2A, blue circles), indicative of cell death (S1 Fig). Rapid death of *lys*^−^ cells during lysine starvation was prevented by rapamycin—an inhibitor of the growth activator TORC1 (Fig 2A, green crosses)—or by removing the growth activator glucose (Fig 2A, green squares). The viability of lysine-starved *lys*^−^ cells was further reduced by deleting *BCY1*, an inhibitor of the Ras/PKA growth pathway (Fig 2A, red diamonds; S2 Fig; Fig 1A).

Consistent with nutrient–growth dysregulation, autophagy is reduced in *lys*^−^ cells during lysine limitation compared to natural limitation. We used a GFP-Atg8 cleavage assay [28] to monitor autophagy. Specifically, when cells undergo autophagy, GFP-Atg8 is delivered to the vacuole and cleaved, but the free GFP has high resistance to vacuolar degradation. Thus, high ratio of GFP/GFP-Atg8 indicates high autophagy activity [29]. *lys*^−^ cells starved for lysine showed significantly less autophagy activity compared with *lys*^−^ cells deprived of the natural nutrient nitrogen or simultaneously starved for nitrogen and lysine (Fig 2B).

In summary, *lys*^−^ cells limited for lysine suffer nutrient–growth dysregulation manifested as reduced viability and autophagy compared to during natural limitation.

### Repeated evolution of organosulfur auxotrophy

To examine how cells might cope with nutrient–growth dysregulation, we evolved *lys*^−^ cells in lysine limitation for tens of generations, either as monocultures in lysine-limited chemostats or in cocultures with a lysine-releasing strain (Methods, “Evolution”; “Chemostats and turbidostats”). We then randomly chose a total of approximately 50 evolved *lys*^−^ clones for whole-genome sequencing. Similar to our previous findings [30,31], each clone carried at least 1 mutation that increased the cell’s affinity for lysine (S2 Table). These mutations included duplication of chromosome 14, which harbors the high-affinity lysine permease gene *LYP1* [32], or loss of or reduction in activities of genes involved in degrading Lyp1 (e.g., *ECM21* [extracellular mutant gene 21], *RSP5* [reverses spt- phenotype gene 5], and *DOA4* [degradation of alpha gene 4]) [31,33].

**Fig 3:**
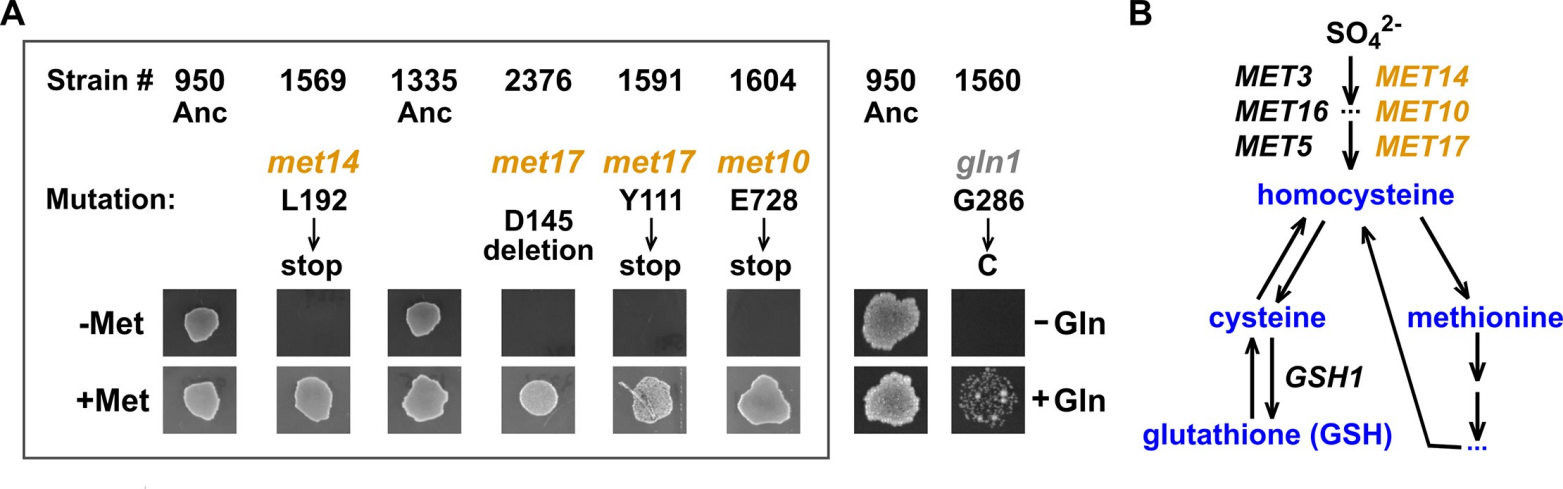
The evolution of auxotrophy. (A) The evolution of auxotrophs. Ancestral *lys*^−^ cells (WY950; WY1335) were grown for tens of generations in minimal medium, either in lysine-limited chemostats [35] or via coculturing with a lysine releaser in a cross-feeding yeast community [30,36]. Out of 20 independent lines, we randomly isolated approximately 50 clones for whole-genome sequencing. Chemostat evolution and coculture evolution both yielded *met*^−^ mutants: 1 (WY1604) out of 9 clones in chemostat evolution; 3 (WY1569, WY2376, WY1591) out of 42 clones in coculture evolution). These mutants, all isolated from independent lines, required an externally supplied organosulfur such as methionine to grow. A glutamine auxotrophic *gln1* clone was also identified. In the experiment shown here, clones were grown to exponential phase in SD supplemented with amino acids, washed with SD, starved for 3 hours to deplete cellular storage, and spotted on indicated agar plates at 30°C. (B) The organosulfur synthesis pathway in *S*. *cerevisiae*. *S*. *cerevisiae* utilizes sulfate supplied in the medium to synthesize the organosulfur homocysteine, which is then used to make a variety of other organosulfurs, including methionine, cysteine, and GSH [37]. All *orgS*^−^ mutants we have identified (orange) fail to synthesize homocysteine, and thus can be supported by any of the organosulfurs depicted here (blue; note the interconvertibility between organosulfurs). Anc, ancestor; *met*^−^, methionine-requiring mutant; *gln1*, glutamine metabolism gene 1 mutant; GSH, reduced glutathione; SD, synthetic minimal glucose medium.

Surprisingly, even though the input medium contained no organosulfurs (sulfur-containing organic molecules; blue in Fig 3B), approximately 10% of the 51 sequenced clones harbored mutations in the biosynthetic pathway that converts externally supplied sulfate to essential organosulfurs. These *met*^−^ (methionine-requiring) mutations included *met10*, *met14*, and *met17* (Fig 3A, box) and arose from independent chemostat and coculture lines (S2 Table). Indeed, these mutant clones required an external source of organosulfur such as methionine to grow (Fig 3A, box) [34]. We designate these mutants as *lys*^−^*orgS*^−^. *lys*^−^*orgS*^−^ arose after the initial evolutionary adaptation to lysine limitation, since *lys*^−^ and *lys*^−^*orgS*^−^ clones from the same culture harbored an identical *ecm21* or *rsp5* mutation (S2 Table, matched color shading). We also found a glutamine auxotroph *gln1* (Fig 3A, see Discussions). Since we observed *gln1* only once, we focused on the evolution of *orgS*^−^.

When we tested 6 independently evolved cultures (3 chemostat monocultures and 3 cross-feeding cocultures) at approximately 30–80 generations (using the growth assay in Fig 3A), *lys*^−^*orgS*^−^ mutants could be detected in all cases (S3 Fig; Methods, “Quantifying auxotroph frequency”), suggesting repeated evolution of organosulfur auxotrophy. Below, we focus on *lys*^−^ monocultures because in these cultures, *lys*^−^*orgS*^−^ cells must have received organosulfurs from *lys*^−^ cells.

### The organosulfur niche mainly consists of glutathione S-conjugates and glutathione

**Fig 4:**
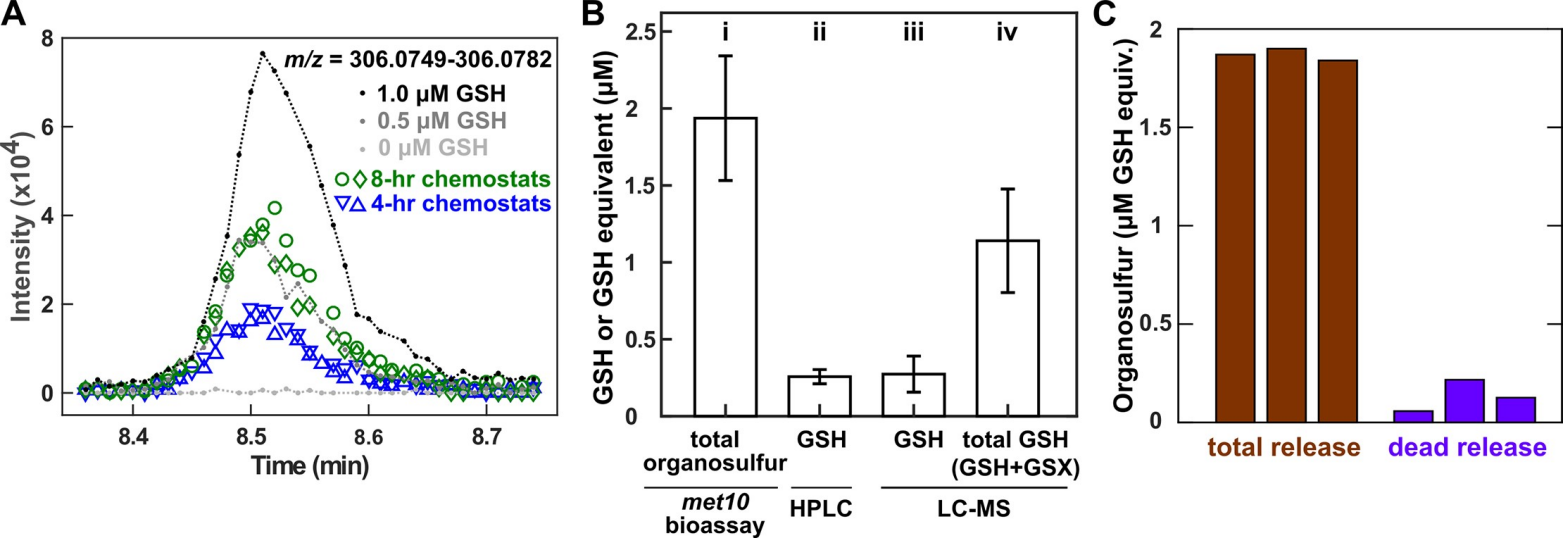
Lysine-limited *lys*^−^ cells mainly release GSH and GSXs. Ancestral *lys*^**−**^ cells (WY1335) were cultured in lysine-limited chemostats at 8-h doubling time unless otherwise indicated. (A) Mass spectra traces of GSH ion. Gray and black lines correspond to known quantities of GSH added to fresh growth media. Blue and green correspond to filtered supernatants harvested at approximately 48 hours from chemostats at 4-hour and 8-hour doubling time, respectively, with different symbols denoting replicate chemostats. Plotted data are provided as S2 Data. (B) GSH and GSXs constitute the majority of the organosulfur niche. Supernatants were harvested at steady state cell density (approximately 26 hours) and filtered. (i) Bioassay quantification of total organosulfur was performed by comparing the final turbidity of *met10*^**−**^ (WY1604) grown in supernatants versus in various known concentrations of GSH. (ii, iii) GSH in supernatant was quantified by HPLC and LC–MS. (iv) GSH + GSX in supernatants were quantified by first reducing GSX to GSH with TCEP and then measuring total GSH via LC–MS. Error bars mark 2 standard deviations of samples from 3 independent chemostats. Plotted data are provided as S4 Data. (C) Organosulfurs are likely released by live cells. Total organosulfur in chemostat supernatant (brown) far exceeded that expected from release by dead cells (purple). Organosulfurs were quantified using the *met10* bioassay. To estimate dead-cell release, we measured dead-cell density using flow cytometry (Methods, “Flow cytometry”), and multiplied it with the average amount of organosulfur per cell (Methods, “Metabolite extraction”). Three independent experiments are plotted (S5 Data). GSH, reduced glutathione; GSX, glutathione S-conjugate; HPLC, High-Performance Liquid Chromatography; LC–MS, liquid chromatography–mass spectrometry; *met*^**−**^, methionine-requiring mutant; TCEP, tris(2-carboxyethyl)phosphine.

We initially tested whether chemostat supernatants of lysine-limited *lys*^−^ cells contained methionine or cysteine using gas chromatography. Both compounds were undetectable. We then resorted to liquid chromatography–mass spectrometry (LC–MS) (Methods, “LC–MS”). We identified reduced glutathione (GSH) as a released organosulfur (Fig 4A; S4 Fig and S5 Fig). GSH, a tripeptide comprising glutamate, cysteine, and glycine, is a major cellular redox buffer and can form GSH-S-conjugates (GSXs) with itself (GS-SG) and with other compounds via disulfide bonds (e.g., GS-S-Coenzyme A [CoA]) [38]. Indeed, *gsh1*^−^ cells, whose growth can be supported by externally supplied GSH and GS-SG, but not methionine (S5B Fig) [39], grew in chemostat supernatants (S5E Fig). GSH release rate was comparable between ancestral and evolved *lys*^−^ cells in lysine-limited chemostats (S7D Fig), and thus, organosulfur supply was uninterrupted during evolution.

To estimate the total organosulfur niche in chemostat supernatant, we developed a yield-based bioassay (Methods, “Bioassays”). We used *met10*^−^, a mutant isolated in our evolution experiment that can use a variety of organosulfurs, including GSH, GS-SG, and methionine (S5C and S5D Fig). By comparing the final turbidity yield of *met10*^−^ in a chemostat supernatant versus in minimal medium supplemented with various known amounts of GSH, we can estimate supernatant organosulfurs in terms of GSH equivalents (Fig 4B, i). GSH quantified via High-Performance Liquid Chromatography (HPLC) and LC–MS (Methods, “Analytical chemistry quantification of GSH and GSXs”) was much lower than the total organosulfur niche (Fig 4B, compare ii and iii with i). We then chemically reduced chemostat supernatants to convert GSX to GSH and measured total GSH via LC–MS (Methods, “LC–MS”). We found that GSH and GSX together dominated the organosulfur niche (Fig 4B, compare i and iv), with GSX being the major component (Fig 4B, compare iii with iv).

The organosulfur niche was mainly created by live-cell release rather than by dead-cell lysis. Specifically, we compared the organosulfur concentration quantified in chemostat supernatant (Fig 4C, brown) with that expected from dead-cell release (calculated by multiplying dead-cell density with average intracellular organosulfur content; Methods “Metabolite extraction”) (Fig 4C, purple). Our measurements suggest that organosulfurs were mainly released by live cells (Fig 4C; S6 Fig), consistent with the observation that GSH and GSX are exported from the cell in an ATP-dependent fashion [38,40].

### Organosulfur release is associated with nutrient–growth dysregulation, not slow growth

To test whether organosulfur release is associated with nutrient–growth dysregulation, we grew *lys*^−^ cells in turbidostats [41] (Methods, “Chemostats and turbidostats”) in excess lysine where nutrient–growth regulation should be normal. *lys*^−^ cells in excess lysine released GSH at a significantly slower rate than in lysine-limited chemostats (S7A–S7C Fig).

**Fig 5:**
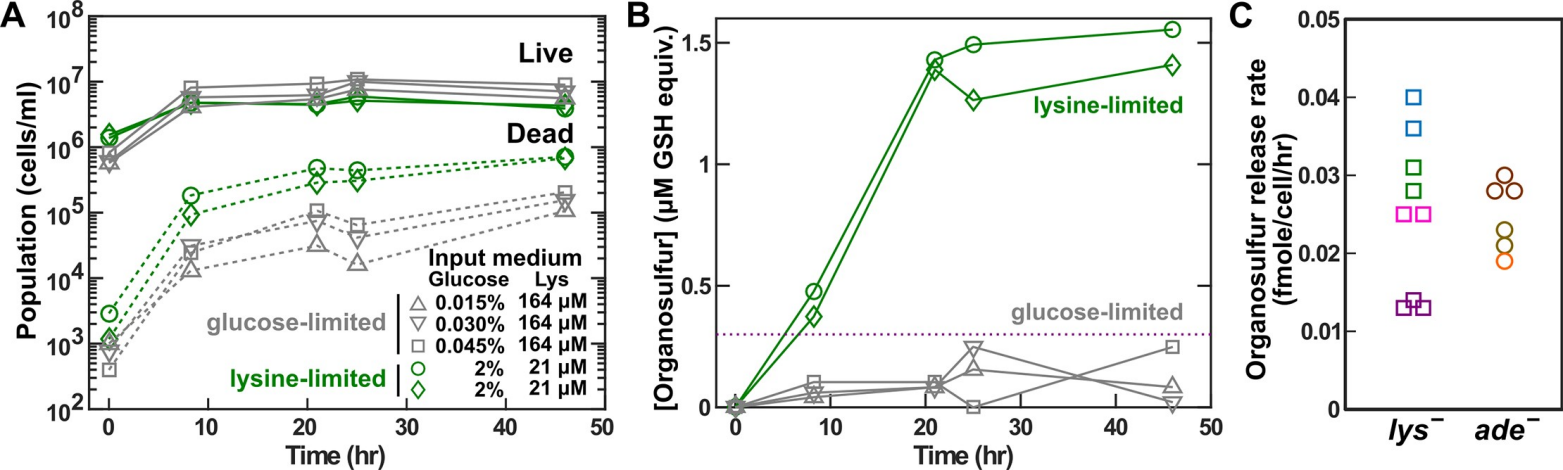
Organosulfur release is associated with nutrient–growth dysregulation. *lys*^−^ cells (WY1335) were cultured in lysine-limited (green) or glucose-limited (gray) chemostats at 8-hour doubling time. The concentrations of glucose and lysine in the input fresh minimal medium are marked. (A) Higher percentage of dead cells in lysine-limited chemostats than in glucose-limited chemostats. Live- and dead-cell densities were measured via flow cytometry (Methods, “Flow cytometry”; S6 Data). (B) Higher organosulfur concentrations in lysine-limited chemostats than in glucose-limited chemostats. Organosulfur concentration was measured in terms of GSH equivalents using the *met10* bioassay (S5 Fig), with dotted line marking the lower limit of the linear detection range (S6 Data). (C) Adenine-limited *ade*^−^ cells release organosulfurs at comparable rates as lysine-limited *lys*^−^ cells. Cells were cultured in chemostats limited for the respective auxotrophic metabolite at 8-h doubling time until a steady state was reached. Release rate was quantified using *r* = *dil* × [orgS]_*ss*_/[Live]_*ss*_ (Eq 14 in [30]), where *dil* is the dilution rate (*ln*2/8/hour), [orgS]_*ss*_ is the steady-state organosulfur concentration (measured in terms of GSH equivalents using the *met10* bioassay; S5 Fig), and [Live]_*ss*_ is the steady-state live-cell density. Different colors correspond to experiments done on different days, and each data point represents an independent chemostat (S5 Data). *ade*^−^, adenine-requiring mutant; GSH, reduced glutathione; *lys*^−^, lysine-requiring mutant; *met*, methionine-requiring mutant.

To further test whether organosulfur release is associated with nutrient–growth dysregulation or with slow growth, we compared *lys*^−^ cells in glucose-limited chemostats versus lysine-limited chemostats at the same doubling time. Nutrient–growth regulation should be normal during glucose limitation, but not during lysine limitation (Fig 1B). Indeed, the percentage of dead cells was much lower during glucose limitation than during lysine limitation (Fig 5A; note the logarithm scale). Organosulfurs accumulated to a steady state in lysine-limited chemostats but were barely detectable in glucose-limited chemostats, even though live population density was higher in glucose-limited chemostats (Fig 5A and 5B).

Organosulfurs were also released by nutrient–growth-dysregulated *ade*^−^ cells in adenine-limited chemostats. Adenine-limited *ade*^−^ cells released organosulfurs at a similar rate as lysine-limited *lys*^−^ cells (Fig 5C), and this release was mediated by live cells (S8 Fig). Although differing from lysine-limited *lys*^−^ cells in several aspects (S9 Fig), adenine-limited *ade*^−^ cells displayed nutrient–growth dysregulation: *ade*^−^ cells lost viability during adenine starvation, and this low viability was rescued by removing glucose (S9 Fig).

Taken together, these results suggest that organosulfur release is associated with nutrient–growth dysregulation, not with slow growth.

### Organosulfur limitation confers a frequency-dependent fitness advantage to *orgS*^−^

*lys*^−^*orgS*^−^ repeatedly rose from 1 mutant cell to a detectable frequency in independent cultures (approximately 10% of 50 randomly selected clones from 20 independent lines; S2 Table). This suggests that the *orgS*^−^ mutation may confer a fitness benefit to *lys*^−^ cells. To test this, we randomly chose an evolved *lys*^−^*orgS*^−^ clone (WY1604), restored its *orgS*^−^ (*met10*) mutation to wild type, and compared isogenic *lys*^−^*orgS*^−^ and *lys*^−^ clones in a variety of nutrient environments.

**Fig 6:**
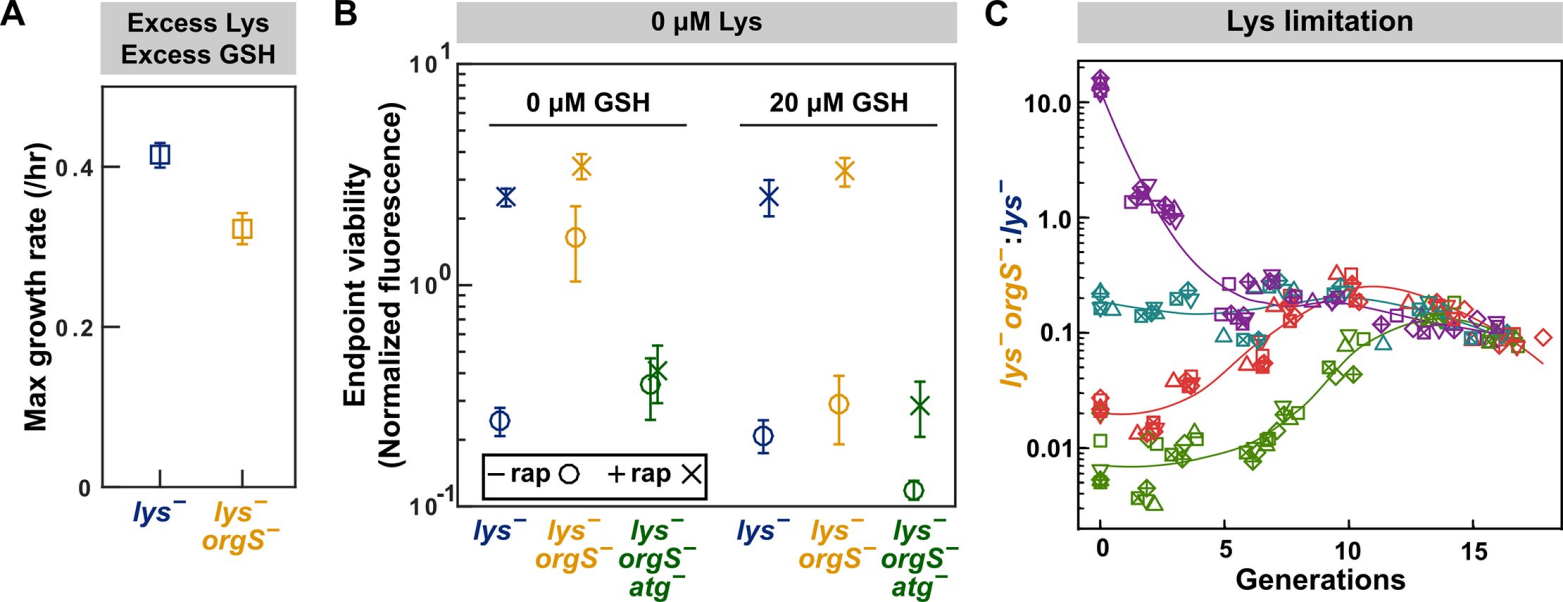
*lys*^−^*orgS*^−^ displays a frequency-dependent fitness advantage over *lys*^−^ during lysine limitation if organosulfur is also limited. (A, B) Fitness advantage of *lys*^−^*orgS*^−^ over *lys*^−^ requires low organosulfur and autophagy. Cell growth was imaged via fluorescence microscopy. (A) In excess lysine and GSH conditions, *lys*^−^*orgS*^−^ (WY1604, orange) grew slower than isogenic *lys*^−^ (WY2429, blue). All data can be found in S7 Data. (B) Isogenic *lys*^−^ (WY2429, blue), *lys*^−^*orgS*^−^ (WY1604, orange), and *lys*^−^*orgS*^−^*atg5*^−^ (WY2370, green) were cultured in the absence of lysine without (circles) or with (crosses) 1 μM rapamycin and without or with 20 μM GSH. Total fluorescence of the field of view at the endpoint (110 hours) was normalized against that of time zero. *lys*^−^*orgS*^−^ (yellow circle, second column) survived lysine starvation better than *lys*^−^ (blue circle, first column) when GSH was low. When GSH was abundant, *lys*^−^*orgS*^−^ (yellow circle, fifth column) behaved similarly to *lys*^−^ (blue circle, fourth column), surviving lysine starvation poorly and rescued by the growth inhibitor rapamycin (yellow and blue crosses, fifth and fourth columns). The high viability of organosulfur-limited *lys*^−^*orgS*^−^ and of rapamycin-treated cells were abolished when autophagy was prevented (WY2370, green circles and crosses). Full data are plotted in S10 Fig and provided as S8 Data. Error bars represent 2 standard deviations from 4 positions in a well. (C) Negative-frequency–dependent fitness advantage of *lys*^−^*orgS*^−^ over *lys*^−^. Isogenic BFP-tagged *lys*^−^*orgS*^−^ (WY2072 or WY2073) and mCherry-tagged *lys*^−^ (WY2045) were placed in competition in a lysine-limited environment by coculturing with a lysine-releasing strain (WY1340) (Methods). The ratio of *lys*^−^*orgS*^−^ to *lys*^−^ over time was measured by flow cytometry (Methods, “Competition”). Smooth lines serve as visual guide. Different colors represent different starting ratios, while different symbols represent independent experiments. Plotted data can be found in S9 Data. *atg5*, autophagy-related gene 5 mutant; GSH, reduced glutathione; *lys*^−^, lysine-requiring mutant; *orgS*^−^, organosulfur-requiring mutant; rap, rapamycin.

According to the prevalent “energy-saving” hypothesis [42,43], excess organosulfurs should help *lys*^−^*orgS*^−^ by sparing it the cost of de novo synthesis of organosulfurs. However, we observed the opposite: when both GSH and lysine were in excess, *lys*^−^*orgS*^−^ grew significantly slower than *lys*^−^ (Fig 6A). When GSH was in excess but lysine was absent, *lys*^−^*orgS*^−^ behaved similarly to *lys*^−^, surviving poorly but regaining viability when growth was inhibited by rapamycin (Fig 6B, right, compare orange with blue; S10B Fig).

We then tested an alternative hypothesis: unable to process sulfate in the medium but able to utilize organosulfurs released by *lys*^−^, *lys*^−^*orgS*^−^ cells may respond to this organosulfur limitation similarly as to a natural limitation, mounting stress responses including autophagy. This might in turn confer *lys*^−^*orgS*^−^ a fitness advantage over nutrient–growth-dysregulated *lys*^−^ cells. Indeed, when organosulfur was limiting, *lys*^−^*orgS*^−^ survived lysine limitation better than *lys*^−^ (Fig 6B, left, orange circle higher than blue circle; S10A Fig). A similar trend was observed at intermediate levels of lysine and GSH (S11 Fig). We would ideally like to compare autophagy activities between *lys*^−^*orgS*^−^ and *lys*^−^ cells, but there are multiple technical challenges (see S12 Fig legend for an explanation). In a less ideal comparison, *lys*^−^*orgS*^−^ showed lower autophagy activity during lysine starvation than during either organosulfur starvation or organosulfur/lysine double starvation (S12 Fig). Importantly, deletion of *ATG5*, a gene essential for autophagy, diminished the advantage of *lys*^−^*orgS*^−^ over *lys*^−^ during lysine limitation (Fig 6B, orange symbols higher than green symbols; S10 Fig).

To directly test any fitness difference between isogenic *lys*^−^ and *lys*^−^*orgS*^−^ clones, we marked them with different fluorescent proteins and placed them in competition in lysine limitation (Methods, “Competition”). When *lys*^−^*orgS*^−^ was initially abundant (Fig 6C, purple), its frequency declined as expected because of competition for the limited organosulfurs released from rare *lys*^−^. When *lys*^−^*orgS*^−^ was rare, its frequency initially increased (Fig 6C, red and green), demonstrating a fitness advantage over *lys*^−^. Regardless of the starting point, the ratio of *lys*^−^*orgS*^−^/*lys*^−^ converged to a steady-state value (Fig 6C).

The observed steady-state ratio of *lys*^−^*orgS*^−^/*lys*^−^ is consistent with organosulfur release and consumption measurements. At steady-state species ratio, organosulfur releasers and consumers must grow at an identical rate, and organosulfur concentration is at a steady state. Assume that all organosulfur compounds are consumable by *lys*^−^*orgS*^−^ as GSH equivalents. The organosulfur concentration *O* at steady state can be described as $\frac{dO}{dt}=rR-cgC$, where *R* and *C* are the concentrations of releaser and consumer cells, respectively; *r* represents the release rate of organosulfurs per releaser cell; *g* represents growth rate (= dilution rate in chemostats); and *c* represents the amount of organosulfur consumed per consumer birth. *r*, the organosulfur release rate by *lys*^−^ in 8-hour chemostats (*g* = *ln*2/8/hour), was approximately 0.02 fmole GSH equivalent/cell/hour (Fig 5C). From the organosulfur-OD standard curve (e.g., S5 Fig, blue curves; 1 OD is approximately 3 × 10^7^ cells/ml in our setup), organosulfur consumption per birth *c* is approximately 2 fmole GSH/cell. Setting the above equation to zero, we obtain $\frac{C}{R}=\frac{r}{cg}=\frac{0.02\;fmole/cell/hr}{(2\;fmole/cell)*(ln2/8hr)}$ approximately 0.1, consistent with the observed ratio of approximately 0.1 (Fig 6C).

In summary, by helping restore nutrient–growth regulation (at least partially), organosulfur limitation confers rare *lys*^−^*orgS*^−^ a frequency-dependent fitness advantage over *lys*^−^ during lysine limitation (S13 Fig).

## Discussion

### Nutrient–growth dysregulation and the evolution of metabolic dependence

Our work demonstrates that within tens of generations, lysine-limited *lys*^−^ cells often evolved into 2 subpopulations: *lys*^−^ and *lys*^−^*orgS*^−^ (Fig 3 and S3 Fig). *lys*^−^ cells initially adapted to lysine limitation via mutations such as *ecm21*, *rsp5*, and *DISOMY14* (duplication of chromosome 14). Increased lysine permease Lyp1 on the cell membrane enabled these mutants to outcompete ancestral cells in lysine-limited environments [30,32,31].

**Fig 7:**
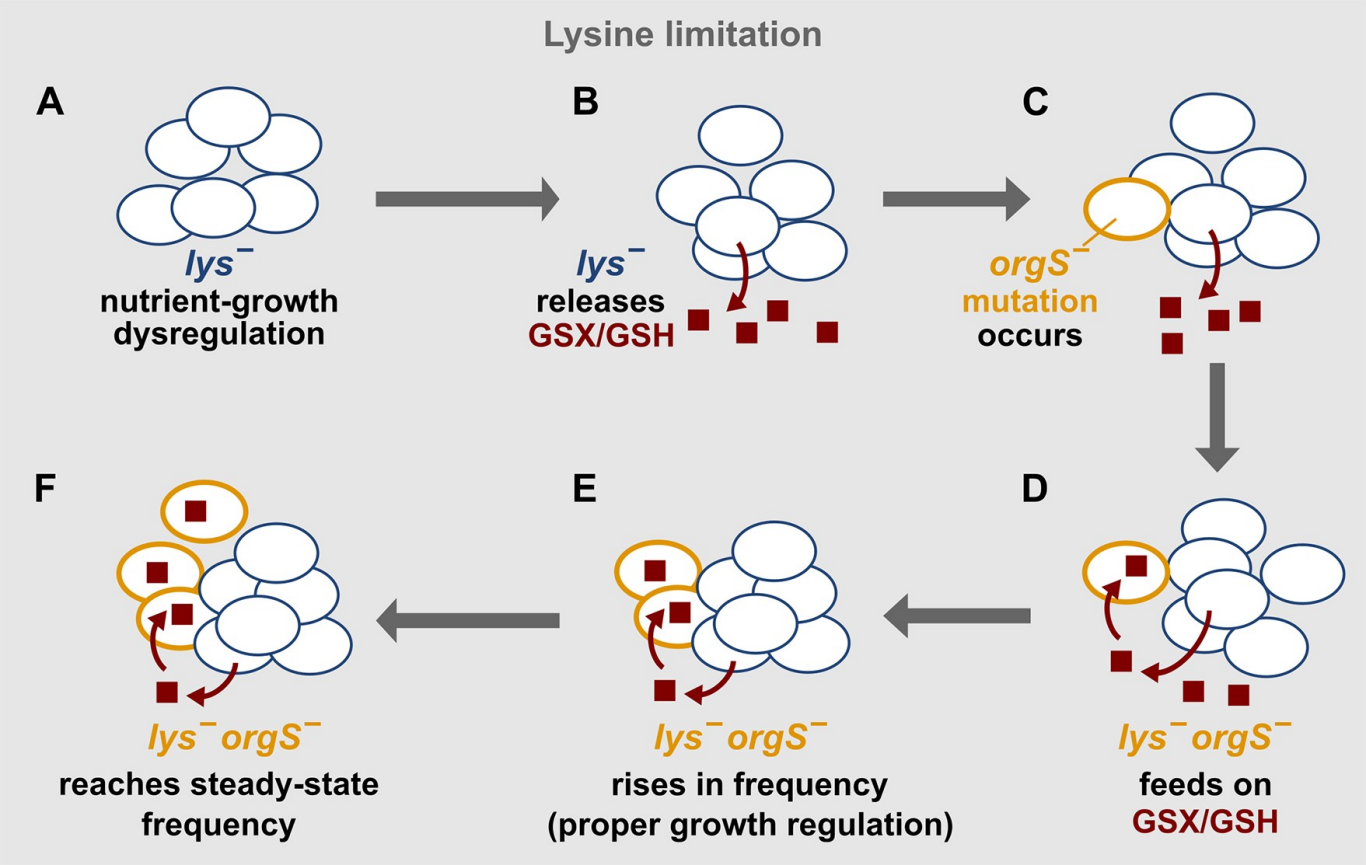
Nutrient–growth dysregulation and the evolution of an organosulfur-mediated interaction. (A) During lysine limitation, *lys*^−^ cells suffered reduced autophagy and viability compared to during natural limitation (Fig 2). (B) These cells released organosulfurs mainly comprising GSX and, to a lesser extent, GSH (Fig 4). We hypothesize that organosulfur release is a detoxification response to nutrient–growth dysregulation because organosulfur release was significantly reduced during glucose limitation (Fig 5B) or in excess lysine (S7 Fig). (C–D) The released organosulfurs created a metabolic environment that could support the growth of the newly arisen *lys*^−^*orgS*^−^. (E) The rare *lys*^−^*orgS*^−^ mutant rose in frequency (Fig 6C) because of a fitness advantage gained by properly responding to sulfur limitation—a natural limitation; e.g., compared with lysine-starved *lys*^−^ cells, *lys*^−^*orgS*^−^ cells doubly starved for sulfur and lysine maintained high cell viability in an autophagy-dependent fashion (Fig 6B, left). (F) Eventually, *lys*^−^*orgS*^−^ reached a steady-state frequency at which organosulfur supply and consumption were balanced (Fig 6C). GSH, reduced glutathione; GSX, glutathione-S-conjugate; *lys*^−^, lysine-requiring mutant; *orgS*^−^, organosulfur-requiring mutant.

After this initial adaptation to lysine limitation, 2 aspects of nutrient–growth dysregulation encouraged the evolution of *orgS*^−^ (Fig 7). First, live lysine-limited *lys*^−^ cells released organosulfurs consisting primarily of GSX and, to a lesser extent, GSH (Fig 4; S4 Fig, S6 Fig and S7 Fig). This created an organosulfur niche for the survival of *lys*^−^*orgS*^−^ (S5 Fig). Second, when *lys*^−^*orgS*^−^ initially arose, the mutant enjoyed a fitness advantage over *lys*^−^ (Fig 6C; S13 Fig).

The fitness advantage of *lys*^−^*orgS*^−^ over *lys*^−^ is presumably due to *lys*^−^*orgS*^−^ properly sensing and responding to sulfur limitation. Indeed, autophagy and low organosulfur were required for the enhanced viability of *lys*^−^*orgS*^−^ compared with *lys*^−^ cells during lysine limitation (Fig 6B and S11 Fig). Consistent with this notion, mutants defective in methionine synthesis died slowly during methionine limitation [44]. Furthermore, methionine auxotrophs (e.g., *met6* or *met13*) responded to methionine starvation by engaging in oxidative metabolism, similar to how cells respond to phosphate or sulfur starvation [6] (Fig 1B, red box). As *lys*^−^*orgS*^−^ cells increased in frequency, their fitness advantage over *lys*^−^ diminished because of competition for organosulfurs released by *lys*^−^ cells (Fig 6C and S13 Fig). Eventually, a steady-state ratio of *lys*^−^*orgS*^−^/*lys* ^−^ was established (Fig 6C).

The long-term dynamics of *orgS*^−^ is likely complex, especially in environments with fluctuating nutrient availability. Although *lys*^−^*orgS*^−^ enjoyed a frequency-dependent fitness advantage over *lys*^−^ during lysine limitation (Fig 6C), *lys*^−^*orgS*^−^ suffered a fitness disadvantage in excess lysine and GSH (Fig 6A). Thus, *lys*^−^*orgS*^−^ can go extinct during nutrient excess, although during lysine limitation, new *orgS*^−^ mutants can arise and increase in frequency to a steady-state ratio. In any environment, if additional mutations acquired by *lys*^−^ happened to be much more adaptive than those acquired by *lys*^−^*orgS*^−^, then *lys*^−^*orgS*^−^ can become very rare or even go extinct, similar to [31].

We also recovered a *gln1*^−^ mutation in *lys*^−^ cells evolving in lysine limitation (Fig 3A). Thus, lysine-limited *lys*^−^ cells must have released glutamine or glutamine derivatives that supported *lys*^−^*gln*^−^ cells. Intriguingly, glutamine is the key limiting intracellular metabolite upon nitrogen limitation in *S*. *cerevisiae* [15]. Thus, *lys*^−^*gln*^−^ might properly down-regulate growth by sensing nitrogen limitation [2,10], gaining a frequency-dependent fitness advantage over *lys*^−^ cells. The glutamine auxotroph was much rarer than organosulfur auxotrophs, possibly because glutamine consumption per cell is large with respect to glutamine release rate. A future direction would be to characterize metabolites released by cells under unnatural versus natural nutrient limitations and to understand why metabolites are released and how released metabolites affect other cells.

### GSX and GSH release: How and why?

GSH is the major cellular redox buffer in eukaryotes. GSH carries out a variety of functions such as protecting cells against stresses, storing and transporting sulfur, and detoxifying metals and xenobiotics [45]. Toxic metals and xenobiotics are conjugated to glutathione in the form of GSX and subsequently exported from cells, and often GSH is coexported [45]. Stresses such as temperature shock, nitrogen depletion, and progression into stationary phase induce genes encoding glutathione precursor synthesis, glutathione S-transferase, and glutathione peroxidase [14]. Thus, glutathione detoxification is an integral part of responding to stresses including nutrient limitation.

We propose that nutrient–growth dysregulation can trigger the release of organosulfurs. Our proposition is consistent with the following observations. First, organosulfurs are barely released by *lys*^−^ cells grown in excess lysine (S7 Fig) but rapidly released by live cells upon lysine limitation (Figs 4C and 5B). In fact, the more severe the lysine limitation, the higher the organosulfur release rate (S4C Fig). Second, organosulfur release is not just a consequence of slow growth. At the same slow growth rate, glucose-limited *lys*^−^ cells (under proper nutrient–growth regulation) released organosulfurs at a much lower level than lysine-limited *lys*^−^ cells (Fig 5B). Third, adenine-limited *ade*^−^ cells, which suffered aspects of nutrient–growth dysregulation (S9 Fig) [27], also released a significant amount of organosulfurs (Fig 5C). Together, these results suggest that nutrient–growth dysregulation is associated with, and can possibly trigger, organosulfur release.

How might organosulfurs be released? Cellular GSX and GSH are likely released by live cells rather than leaked from dead cells (Fig 4C; S6 Fig and S8 Fig), consistent with the ATP-dependent export of GSH and GSX previously observed in yeast [40]. We hypothesize that during nutrient–growth dysregulation, GSH conjugates with cellular targets and GSXs were released for detoxification. One possibility is that auxotrophic cells limited for a specific metabolite may futilely up-regulate the biosynthesis of that metabolite, and because biosynthesis often involves redox reactions, cells could suffer redox imbalance. In this case, glutathione detoxification may be employed to maintain a viable redox state in the cell. GSX/GSH release is presumably mediated by one or several GSX/GSH efflux pumps, including Opt1 (oligo peptide transporter protein 1), Gex1 (glutathione exchanger 1), Gex2 (glutathione exchanger 2), and Gxa1 (glutathione export ABC protein 1). Organosulfur release may also occur via exocytosis of vacuolar GSX/GSH, after organosulfurs have been transported from cytoplasm to vacuole via, e.g., Ycf1 (yeast cadmium factor protein 1) and Bpt1 (bile pigment transporter protein 1) [45]. Note that some of these transporters are known to be upregulated during stresses [6,46]. A low level of GSH is coexported with GSX either because GSH can serve as a low-affinity substrate for GSX exporters or because GSH export can facilitate GSX export [45]. Future molecular identification of the released GSX conjugates will shed light on how glutathione and nutrient–growth dysregulation might be linked.

Why might GSX and GSH be released from cells suffering nutrient–growth dysregulation? We present 2 opposite hypotheses on the fitness consequences of organosulfur release. In the first hypothesis, organosulfur release incurs no cost to self and may even provide benefits. Redox buffering via GSH conjugation and the subsequent export of GSX is thought to be vital for detoxification and defense against oxidative stress [47,48]. Moreover, extracellular GSH may help cells maintain membrane integrity [49] and survive adverse environments [50]. In the second hypothesis, GSH/GSX over-release may be costly to the releaser and occurs as a pathology to *lys*^−^ cells under unnatural limitation. Both the production and transport of GSH are energy-intensive processes [40,51]. Moreover, GSH production involves a considerable “reduction cost” [37,52]. To test whether GSH/GSX release is costly, one could block release by removing all relevant transporter genes and then compare the fitness of this mutant with that of the parent strain.

### Diverse routes to the evolution of metabolic interactions

Chemical release by cells (“niche construction”) mediates diverse microbial interactions [53–56]. Often, metabolites are released as a consequence of “overflow” metabolism [57–61]; e.g., yeast excretes amino acids as a consequence of nitrogen overflow, and the released amino acids in turn enable the survival of symbiotic lactic acid bacteria [58]. As another example, when yeast increases potassium uptake during potassium limitation, ammonium—of similar size as potassium—leaks into the cell. Because high ammonium influx is toxic, the cell detoxifies ammonium by excreting amino acids [62].

Auxotrophs are predicted to be widespread [42,63] and have been found in natural isolates [64–69]. When nutrients are supplied by either the medium or other microbes, initially rare auxotrophic mutants can rise to a detectable frequency [42,43,70,71] via diverse mechanisms. First, at a high mutation rate (e.g., a high loss rate of plasmids that carry biosynthetic genes), auxotrophs can rise to a detectable level [72] via mutation-selection balance: a high level of mutant is generated, and a fraction is purged by selection. Second, an auxotrophic mutation can rise to a high frequency if it happens to occur in a highly fit genetic background (“genetic hitchhiking”). Third, an auxotroph can have a fitness advantage over its nonauxotrophic (“prototrophic”) counterpart. The “energy-saving” hypothesis posits that auxotrophs may gain a fitness advantage over prototrophs by saving the energy of synthesizing the essential nutrient [43]. Contrary to the energy-saving hypothesis, the fitness advantage of *lys*^−^*orgS*^−^ over *lys*^−^ required low organosulfurs (Fig 6; S10 Fig and S11 Fig). The fitness advantage also required autophagy (Fig 6 and S10 Fig), a hallmark of proper growth regulation during nutrient limitation. Overall, limited organosulfurs, by mimicking natural sulfur limitation and thus partially restoring proper nutrient–growth regulation, allowed *lys*^−^*orgS*^−^ cells to survive lysine limitation better than *lys*^−^ (Fig 6).

Interestingly, nutrient–growth dysregulation has been implicated in certain cancers [73]. Thus, understanding the evolutionary and ecological consequences of nutrient–growth imbalance in mammalian cells could be fruitful. In addition, we can view “unnatural limitation” as posing an evolutionarily novel stress that cells are ill-adapted for. We speculate that when encountering evolutionarily novel stresses (e.g., introduced by anthropogenic impacts), microbes may release metabolites that are normally not released, potentially facilitating the evolution of unexpected new ecological interactions.

## Methods

### Medium and strains

All strains used in this study are listed in S1 Table. All yeast nomenclature follows the standard convention. For all experiments, frozen yeast strains stored at −80°C were first struck onto YPD plates (10 g/L yeast extract, 20 g/L peptone, 20 g/L glucose + 2% agar) and grown at 30°C for approximately 48 hours, from which a single colony was inoculated into 3 mL of YPD and grown overnight at 30°C with agitation. All experiments were carried out within 5 days of generating the overnight culture. Minimal medium (SD) contained 6.7 g/L Difco Yeast Nitrogen Base without amino acids but with ammonium sulfate (Thermo Fisher Scientific, Waltham, MA, USA) and 20 g/L D-glucose, except during glucose-limitation experiments, in which lower levels of glucose were used as specified. Glucose starvation medium (S) comprised only 6.7 g/L Difco Yeast Nitrogen Base without amino acids but with ammonium sulfate. Nitrogen starvation medium (SD-N) contained 1.7 g/L Difco Yeast Nitrogen Base without amino acids or ammonium sulfate and 20 g/L D-glucose. Depending on strain auxotrophy, SD was supplemented with lysine (164.3 μM), adenine sulfate (108.6 μM) [74], or organosulfurs (134 μM) so that cells could grow exponentially.

Strains were constructed either via yeast crosses or by homologous recombination [74,75]. Crosses were carried out by mating parent strains, pulling diploids, sporulation, tetrad dissection, and selection on suitable plates. As an example of gene deletion, the *bcy1*Δ strain (WY2527) was constructed by PCR-amplifying the KanMX resistance gene from a plasmid (WSB26 [76]) using the primers WSO671 (TACAACAAGCAGATTATTTTCAAAAGACAACAGTAAGAATAAACGcagctgaagcttcgtacgc) and WSO672 (GAGAAAGGAAATTCATGTGGATTTAAGATCGCTTCCCCTTTTTACataggccactagtggatctg), with a 45-base pair homology (uppercase) to the upstream and downstream region of the *BCY1* gene, respectively. The *lys*^−^ strain WY2490 was then transformed with the PCR product, and transformants were selected on a G418 plate. Successful deletion was confirmed via a checking PCR with a primer upstream of the *BCY1* gene (WSO673 TATACTGTGCTCGGATTCCG) paired with an internal primer for the amplified KanMX cassette (WSO161 ctaaatgtacgggcgacagt).

### Evolution

Coculture evolution was described in an earlier study [32]. To revive a coculture, approximately 20 μL was scooped from the frozen sample using a sterile metal spatula, diluted approximately 10-fold into SD, and allowed to grow to moderate turbidity for 1–2 days. The coculture was further expanded by adding 3 mL of SD. Evolved *lys*^−^ clones were isolated by plating the coculture on rich media (YPD) agar with hygromycin B.

For monoculture evolution, chemostat vessels (S14 Fig) were used (Methods, “Chemostats and turbidostats”). To create a sterile environment, initial assembly was done in autoclave trays, with vessels held in tube racks. Six reservoirs were prepared by adding 810 mL water to each bottle. Six vessels were prepared by adding a 10-mm stir bar and 20 mL growth media (SD + 21 μM lysine) to each vessel. Media delivery tubing was attached between reservoirs and vessels through rubber stoppers, and waste tubing was attached to each outflow arm, with the unattached end covered by foil held in place by autoclave tape. A 1.5-mL microcentrifuge tube was placed over the sampling needle and held in place by autoclave tape. Tubing ports were wrapped with foil as well. Each reservoir with its attached tubing was weighed, the entire assembly autoclaved, then each reservoir weighed again. Lost water was calculated and added back. Under sterile conditions, 90 mL of 10× SD and a lysine stock were added to each reservoir to reach a final lysine concentration of 21 μM. The vessels were then secured into the chemostat manifold receptacles, reservoirs placed on the scales, and tubing threaded into the pumps.

Ancestral or evolved *lys*^−^ clones were grown in 50 mL SD + 164 μM lysine for approximately 20 hours prior to inoculation. Before each experiment, growth was tracked to ensure cells were growing optimally (approximately 1.6-hour doubling time). When cells reached a density of approximately 0.2 OD_600_, cells were washed 3 times in SD and inoculated in a chemostat vessel prefilled with SD + 21 μM lysine. After this step, chemostat pumps were turned on at a set doubling time in the custom-written LabView software package. Each chemostat vessel contained approximately 43 mL running volume and was set to a target doubling time (e.g., for 7-hour doubling, flow rate is 43 × *ln*2/7 = approximately 4.25 mL/h). We evolved 3 lines at 7-hour doubling and 3 lines at 11-hour doubling. With 21 μM lysine in the reservoir, the target steady-state cell density was 7 × 10^6^/mL. In reality, live-cell densities varied between 4 × 10^6^/ml and 1.2 × 10^7^/ml. Periodically, 4 mL of supernatant was harvested and dispensed into a sterile 15-mL conical tube. Next, 300 μL of this cell sample was removed and kept on ice for flow cytometry analysis. The remaining 3.7 mL of supernatant was filtered through a 0.22-μm nylon filter into 500-μL aliquots and frozen at −80°C. Each chemostat was sampled according to a preset sequence. For experiments with metabolite extraction, the chemostat vessel stopper was removed, and cells from 20 mL of sample were harvested (Methods, “Metabolite extraction)”. Because of the breaking of vessel sterility, this would mark the end of the chemostat experiment.

The nutrient reservoir was refilled when necessary by injecting media through a sterile 0.2-μm filter mounted on a 60 mL syringe. To take samples sterilely, the covering tube on the sampling needle was carefully lifted, and a sterile 5-mL syringe was attached to the needle. The needle was then wiped with 95% ethanol and slowly pushed down so that the tip was at least approximately 10 mm below the liquid level. A 5-mL sample was drawn into the syringe, the needle pulled up above the liquid surface, and an additional 1 mL of air drawn through to clear the needle of liquid residue. The syringe was then detached, and the cap was placed back on the needle. The samples were ejected into sterile 13-mm culture tubes for freezing and flow cytometry determination of live-cell densities. In both evolution experiments, samples were frozen in 1 part 20% trehalose in 50 mM sodium phosphate buffer (pH 6.0) + 1 part YPD. The samples were cooled at 4°C for 15 min before being frozen down at −80°C.

Whole-genome sequencing and data analysis are described in detail in [32].

### Quantifying auxotroph frequency

Frozen cultures (2 time points from 3 monoculture evolution and 3 coculture evolution experiments) were revived, and clones were isolated and screened for auxotrophy. We revived frozen samples by directly plating samples on YPD (monoculture) or YPD + hygromycin (cocultures; to select against the partner strain). Plates were grown at 30°C for approximately 2–4 days until all colonies were easily visible for picking. We observed a variety of colony sizes and screened both large and small sized colonies when both were present. We counted large and small colonies to estimate the ratio of large/small colony-forming cells in the population, then multiplied this fraction by the fraction of auxotrophs observed in each colony size class to get a full population auxotroph frequency estimate. To screen for auxotrophy, entire colonies were inoculated into 150 μL of SD, 10 μL of which was diluted into 150 μL SD in microtiter plates and incubated overnight to deplete organosulfur carryover or cellular organosulfur storage. In the case of some small colonies, no dilution was made as the inoculated cell density was already low enough, based on OD measurements. Then, 10–30 μL were diluted into a final volume of 150 μL each of SD + 164 μM lysine, SD + 164 μM lysine + 134 μM methionine, and YPD, aiming for OD of approximately 0.005–0.03 based off an initial reading by a 96-well plate OD_600_ reading of the starvation plate. Plates were then incubated for 48+ hours to grow cultures to saturation, and culture turbidity (OD_600_) was read using a 96-well plate reader. Control wells of known *lys*^−^*orgS*^−^ (WY1604) and *lys*^−^ (WY2226) were included in the screening as controls. Wells that grew in SD + lysine + methionine and YPD but failed to grow in SD + lysine were scored as *lys*^−^*orgS*^−^.

### Fluorescence microscopy

Fluorescence microscopy experiments and data analysis are described in detail elsewhere [27]. Briefly, the microscope is connected to a cooled CCD camera for fluorescence and transmitted light imaging. The microscope harbors a temperature-controlled chamber set to 30°C. The microscope is equipped with motorized switchable filter cubes capable of detecting a variety of fluorophores. It also has motorized stages to allow z-autofocusing and systematic xy-scanning of locations in microplate wells. Image acquisition is done with an in-house LabVIEW program, incorporating autofocusing in bright field and automatic exposure adjustment during fluorescence imaging to avoid saturation. Previous analysis [27] has demonstrated that if fluorescence per cell is constant over time, then background-subtracted fluorescence intensity scales proportionally with live-cell density, and a decrease in fluorescence intensity correlates well with cell death.

For experiments in Fig 2 and S2 Fig, WY2490 (*lys*^−^) and WY2527 (*lys*^−^*bcy1*^−^) were grown overnight to exponential phase in SD + 164 μM lysine, washed 3 times in S medium, and starved for 3 hours at 30°C in factory-clean 13-mm test tubes in either SD (lysine starvation) or S (lysine and carbon starvation). For imaging, approximately 10,000 cells/well were inoculated into each well of a 96-well plate in the corresponding medium (rapamycin treatment was done in SD + 1 μM rapamycin). The microtiter plate was imaged periodically (approximately 1–2 h) under a 10× objective in a Nikon Eclipse TE-2000U inverted fluorescence microscope (Nikon, Tokyo, Japan) using an ET DsRed filter cube (exciter: ET545/30x, emitter: ET620/60m, Dichroic: T570LP). A similar protocol was followed for experiments in Fig 6 and S9, S10 and S11 Figs, with genotypes and starvation conditions noted in the corresponding figure legends.

### Chemostats and turbidostats

Cells were grown under controlled conditions using a custom-made continuous culturing device (S14A Fig), with 6 channels (S14C Fig) of which each can be independently operated as a chemostat or a turbidostat. When operated as a chemostat, a channel provides a limited nutrient environment in which the growth rate is held constant. When operated as a turbidostat, a channel maintains a constant cell density while cells grow with an abundant supply of nutrients. For cell growth protocols in these devices, see Methods, “Evolution”.

The continuous culturing device consisted of 6 reactor vessels (S14A Fig, back), each with a volume of approximately 43 mL (S14E Fig) determined by the height of the outflow tube (S14C Fig). A rubber stopper equipped with an inflow tube and a sampling needle covered the top of each vessel (S14C Fig). The vessels were placed in an aluminum mounting frame with 6 receptacles (S14A Fig, back), each equipped with an integrated magnetic stirrer (made from a CPU fan) and an LED-phototransistor optical detector for OD measurements. The vessels were immobilized in the receptacles by adjustable compression rings. A sampling needle passed through a short length of PharMed rubber tubing (S14C Fig). The tubing was held in place by glass tubing inserted into the stopper. Zip ties are used to achieve the proper tightness, allowing movement of sampling needle while applying enough friction to maintain position. Waste flowed by gravity to a waste receptacle below the device through 0.375-inch inner diameter tubing (Cole Parmer C-Flex) attached to the outflow arm. Nutrient media was fed to each vessel from an independent reservoir by a peristaltic pump (Welco WPM1, operated at 7V DC; Welco, Tokyo, Japan). The media delivery tube consisted of 2 sections of generic 2-mm outer diameter, 1-mm inner diameter PTFE tubing, joined by insertion into the 2 ends of a 17-cm section of PharMed AY242409 tubing (Saint-Gobain, Courbevoie, France) which was inserted into the peristaltic pump (S14B Fig). The pump was activated and deactivated by the custom LabView program through a relay box (Pencom Design, UB-RLY-ISO-EXT-LR; Trumbauersville, PA, USA). Depending on whether a given channel was in chemostat or turbidostat mode, the LabView program controlled the pump in different ways (i.e., constant dilution rate in chemostat, or dilution to a set turbidity in turbidostat). Data for OD and flow rate were logged in for either mode of operation. In both cases, flow rate can be used to calculate growth rate. Media reservoirs (S14A Fig, front) were 1-L glass bottles capped with one-hole rubber stoppers, and a section of glass tubing was used as a sleeve to prevent curling of the PTFE tubing and to keep the end of PTFE tubing touching the bottom of the reservoir. Each reservoir was placed on a digital balance (Ohaus SPX2201; Parsippany, NJ, USA) with a digital interface (Ohaus Scout RS232 interface) for measurement of the volume (weight) remaining in the reservoir at any given time.

When in turbidostat mode, constant average turbidity was maintained. Specifically, the pump was activated when the measured OD was above the set point and deactivated when the OD was below set point. OD was measured using a 940-nm LED (Ledtech UT188X-81-940, driven with 50-ma current; Taiwan, Taiwan) and phototransistor (Ledtech LT959X-91-0125). Each LED-phototransistor pair was tested and selected for consistent OD measurements. The LED and phototransistor were positioned by mounting holes on the aluminum metal frame, on opposite sides of the reactor vessel, 4 cm from the vessel bottom. Each phototransistor was connected to an op-amp (LM324) circuit that acted as a current to voltage converter and buffer (S14B Fig). An isolated DC–DC converter provided a regulated voltage supply for the electronics. The output voltage from the photodetector circuit was digitized using a DAQ (National Instruments USB-6009; Austin, TX, USA) and read by the LabView program for OD measurement. The LabView program stored the average light intensity I_0_ over the first 2 minutes after starting a channel as the “blank” value. The light intensity, I, was measured every approximately 30 s, and the OD = log_10_(I/I_0_) was calculated.

When in chemostat mode, a constant average flow rate *f* of medium into the vessel was maintained. Unlike our earlier chemostat setup [77], here, constant flow rate was achieved via a scale (Ohaus SPX2201) that constantly weighed its associated reservoir, and the reading was acquired through an RS232 interface (Ohaus Scout RS232). The initial scale reading, *M*_*initial*_, was recorded when the chemostat channel was activated or reset. This was used with the current scale reading *M*_*current*_ to calculate the total volume pumped from the reservoir to the vessel as 1 ml/*g* × (*M*_*current*_ − *M*_*initial*_). The target volume that should have flown from the reservoir to the vessel at the current time was calculated according to the preset flow rate. If the total volume was less than the target, the pump was activated, and otherwise, the pump was deactivated. This provided the correct average flow rate. The flow rate was chosen for the desired doubling time, *f* = *ln*(2) × *V*/*T*_*D*_, where *V* is the volume of the vessel and *T*_*D*_ the doubling time. Vessel volumes were calculated by weighing an empty vessel and then weighing it again when filled to the spillover point (S14E Fig), giving an average value of 43 ml. Individual flow rates were determined using individual vessel volumes. Volume measurements are limited by the minimum waste tube drop size of approximately 0.5 ml, which is constrained by surface tension. Scale readings were logged, providing a measure of flow rate (S14D Fig).

### Bioassays

Most bioassays are yield-based. WY1604 (*met10*^−^) usually served as the tester strain unless otherwise specified in an experiment (see S5 Fig and S1 Table), but preparation for all yield-based bioassays was the same. Strains were grown for approximately 16 hours in 3 mL SD, with any required supplements added. During this time, growth rate was tracked to ensure cells were doubling as expected (1.6- to 3-hour doubling depending on the strain/condition). After this time, cells were given 3–5 washes with 3 mL SD + 164 μM lysine (lacking any organosulfur supplements) and starved for at least 3 hours at 30°C in 3 mL SD + 164 μM lysine to deplete cellular reserves of organosulfurs. Starvation was carried out in a factory-clean 13-mm test tube to prevent inadvertent nutrient contamination. Finally, approximately 1,000 cells/well were inoculated in a flat-bottomed 96-well plate into a final volume of 150 μL of either a metabolite standard or a chemostat supernatant, supplemented with SD + 164 μM lysine. For each auxotrophic strain, SD + 164 μM lysine supplemented with various known concentrations glutathione were used to establish a standard curve that related organosulfur concentration (in terms of fmole GSH equivalent) to final turbidity (S5 Fig). Turbidity achieved in a supernatant was then used to infer organosulfur concentration in the supernatant. Plates were wrapped with parafilm to prevent evaporation and incubated at 30°C for 2–3 days. We resuspended cells using a Thermo Fisher Scientific Teleshake (setting #5 for approximately 1 min) and read culture turbidity using a BioTek Synergy MX plate reader (Winooski, VT, USA).

In a rate-based bioassay (S4B Fig), mCherry-tagged yeast strain auxotrophic for organosulfur (WY2035) was pregrown in SD + 164 μM lysine + 134 μM methionine, and growth rate was tracked by optical density to ensure the cell was growing as expected. Next, cells were washed 3 times in SD + 164 μM lysine (lacking organosulfur supplements) and starved for at least 3 hours in factory-clean 13-mm test tubes. OD was measured again, and cells were inoculated to roughly 1,000 cells/well in a 96-well plate in a total volume of 300 μL. The well was filled with either known quantities of organosulfur (methionine or glutathione) or harvested supernatants, both supplemented into SD + 164 μM lysine. The 96-well plate was measured in the same manner as previously outlined in the “Fluorescence microscopy” section.

Maximal growth rate was calculated by measuring the slope of *ln*(Normalized Intensity) against time. For each sliding window of 4 time points, slope is calculated, and if it exceeds the current max slope for the well, it is chosen as the new maximum. To ensure that no estimation occurs when other metabolites such as glucose could be limiting, we restricted analysis to data at 25% maximal intensity to ensure that cells had at least 2 doublings beyond when they are theoretically growing maximally. For rate-based bioassays, maximal growth rates were used to estimate approximate niche size.

### Flow cytometry

Detailed description can be found elsewhere [30]. Population compositions were measured by flow cytometry using a DxP10 (Cytek, Fremont, CA, USA). Fluorescent beads of known concentration (as determined by hemocytometer) were added to determine cell densities. A final 1:20,000 dilution of ToPro3 (Molecular Probes T-3605; Eugene, OR, USA) was used for each sample to determine live- and dead-cell densities. Analysis using FlowJo software showed obvious clustering of live and dead cells in the ToPro3 RedFL1 channel, with dead cells having a RedFL1 signal of >10^3^. Dead-cell densities typically were never higher than 10% in all conditions tested.

### Metabolite extraction

Metabolite extraction for intracellular organosulfur quantification was adapted from [78]. Briefly, 20 mL of chemostat populations was harvested with disposable 25-mL pipettes and rapidly vacuum filtered onto precut 0.45-μm Magna nylon filters (GVS Life Sciences, Sanford, ME, USA). Using ethanol-cleaned forceps, the filter was then quickly submerged into 3 mL ice-cold extraction mixture—40% (v/v) acetonitrile, 40% (v/v) methanol, and 20% (v/v) distilled water—held in a sterile 5-mL centrifuge tube. All reagents were HPLC-grade, and all extraction mixtures were made fresh before each extraction. The centrifuge tube was capped and quickly vortexed to dislodge all cells, and the filter membrane was discarded. The entire process took less than 25 seconds, with the time between populations being filtered and submerged in extraction buffer being less than 10 seconds. After all populations had been harvested, extracts were frozen at −80°C or in liquid nitrogen until solid, transferred to ice, and allowed to thaw. After the samples had thawed, they were incubated on ice for 10 minutes and vortexed once every approximately 3 minutes and returned to −80°C for refreezing (a single “freeze-thaw” cycle). After 3 freeze-thaw cycles, 1.5 mL of sample was harvested and transferred to a new 1.5-mL microcentrifuge tube and centrifuged at 13,000 rpm for 2 minutes at 4°C to pellet the cell debris. The extract was removed, and the remaining cell pellet was extracted again with 1.5 mL of extraction mixture and spun down. The final result was 3 mL of extracted metabolites that was stored at −80°C and analyzed by HPLC less than 48 hours after extraction. For bioassays, this extract was diluted 10-fold in sterile water or SD. To check that a majority of metabolites were extracted, 100 μL of fresh extraction buffer was added to the collected cell debris, vortexed vigorously, and collected by centrifugation. This 100 μL “second extract” was also analyzed for glutathione by HPLC. On average, the amount of glutathione in the second extract was <2% of the amount extracted initially in the 3 mL extraction.

A modified protocol was used for *ade*^−^ cell extracts. We independently verified that the 2 protocols led to comparable results in the *met10* yield-based bioassay (S5 Data). *ade*^−^ chemostat cultures were filtered on 0.45-μm nitrocellulose membranes (Bio-Rad, Hercules, CA, USA), resuspended in extraction mixture, and flash-frozen in liquid nitrogen. Samples were thawed at −20°C over 30 minutes, with vortexing every 5 minutes. Cell debris were pelleted by centrifugation at 13,000 rpm for 10 minutes at 4°C. After the supernatant was transferred to a fresh tube, the pellet was extracted with half the original volume of extraction mixture, and the supernatants from the 2 rounds of extraction were combined. The extraction mixture was dried off using the low temperature setting on a speed-vac, and dehydrated components were resuspended in sterile distilled water.

From the total amount of metabolites in the sample and the total number of cells used to extract metabolites, we can calculate the average amount of metabolite per cell.

### Analytical chemistry quantification of GSH and GSXs

#### HPLC

Reduced glutathione was derivatized using a thiol-specific probe first described by [79], called Thiol Probe IV (EMD Millipore, Burlington, MA, USA) to make a fluorescent glutathione conjugate. The compound reacts readily with free thiols, though at different rates. For quantifying glutathione, 270 μL of sample or GSH standard in SD was added to 30 μL of 833 mM HEPES buffer (pH 7.8). This was done to raise the pH of the sample to a basic level, which facilitates the reaction. Next, the probe (dissolved in DMSO and stored in 50-μL aliquots at −20°C), was added to a final concentration of 100 μM, which is in excess of glutathione by at least 10-fold. The reaction was performed at room temperature in the dark (the probe is light-sensitive) in a 96-well plate for 20 minutes. After this, 8.4 μL of 2M HCl was added to rapidly quench the reaction by lowering the pH to approximately 2. This also stabilizes the fluorescent conjugate. The entire sample was then added to a 250-μL small volume pulled point class insert (Agilent Part No: 5183–2085; Santa Clara, CA, USA) to facilitate autosampler needle access. The small volume insert with sample was then placed inside a dark brown 1.5-mL autosampler vial (Shimadzu part number: 228-45450-91; Kyoto, Japan) and capped with a fresh 9-mm screw cap with PTFE septum (Shimadzu part number: 228-45454-91).

Derivatized glutathione was separated and identified using reverse phase chromatography. 10 μL of the reaction mixture was injected onto a Synergi 4-μM Hydro-RP 80-Å LC Column, 150 × 4.6 mm (Phenomenex, Part No: 00F-4375-E0; Torrance, CA, USA), fitted with a SecurityGuard Cartridges AQ C18 4 x 3.00 mm ID (Phenomenex, Part No: AJO-7511) in a SecurityGuard Cartridge Holder (Phenomenex, Part No: KJ0-4282). The SecurityGuard (precolumn) was periodically replaced whenever pressure reading exceeded the manufacturer’s specifications. Glutathione was eluted from the column with a mobile phase gradient of filtered Millipore water (Solution A) and acetonitrile (Solution B, HPLC-grade). The Millipore water was filtered through a 0.22-μM filter prior to use. Additionally, before each run, the column was equilibrated for 30 minutes with 1% Solution B. The percentage of Solution B followed the following program for each injection: 0 min 1%, 10 min 14%, 10.01 min 1%, and 15 min 1%, corresponding to a gradual increase to 14% Solution B over 10 minutes, followed by a re-equilibration with 1% Solution B. The column was maintained at a running temperature of 25°C in a Nexera X2 CTO-20A oven (Shimadzu). Flow rate was 1 mL /min. Under these conditions, glutathione eluted at approximately 7 minutes, with slight run-to-run variation. Fluorescent glutathione was detected by excitation at 400 nm and emission at 465 nm. After each run, the column was washed and stored per manufacturer’s instructions.

Analysis of HPLC data was done using the R Statistical Language with custom-written software for peak-picking, baseline correction, plotting, and area estimation, which is freely available at https://github.com/robingreen525/Green-PLoSBio-2020-HPLCScripts. Raw data for each sample run were exported to a text file and parsed in the RStudio environment. Emission data (at 465 nm) were culled to restrict analysis from 6.5 to 8 minutes. Next, the script identified a local maximal peak, which, for concentrations above 0.01 μM glutathione, always corresponded to glutathione. Anything lower was indistinguishable from the background. Next, the script identified local minima on both sides of the glutathione peak and drew a baseline that connected the two. The formula (y = mx + b) for this line was calculated, and the baseline was “corrected” by subtracting the emission spectrum value for each point against the y value of the calculated formula for the same point. To quantify the concentration of glutathione in a sample, known concentrations of GSH in SD were subjected to the above procedure. A standard curve of 0.03 to 1 μM was typically used and showed little variability between experiments. A linear regression model of peak area against concentration of reduced glutathione was built using the lm function of the stats package. Samples with areas within the dynamic range (0.03–1 μM GSH) were back-calculated using the linear regression model. Comparing HPLC traces of the same derivatized sample over 24 hours (the approximate time it took to run all samples) shows that glutathione peak area is within 10% for all replicates.

#### LC–MS

Supernatants were shipped overnight on dry ice to the Rabinowitz lab at Princeton University. Stable isotope compound [2-^13^C, ^15^N] GSH was obtained from Cambridge Isotope Laboratories. HPLC-grade water, methanol, and acetonitrile were obtained from Thermo Fisher Scientific. Supernatant sample was thawed at room temperature and 30 μL of the supernatant together with 5 μL of 10 μM 2-^13^C + ^15^N-labeled GSH was transferred into a 1.5-mL centrifuge tube. The samples were either run directly to measure GSH only or first treated with tris(2-carboxyethyl)phosphine (TCEP) to reduce GSX to GSH before measuring total GSH. For those samples with TCEP treatment, 5 μL of 60 g/L TCEP solution (reducing reagent) was added into the sample. The resulting mixture was vortexed and incubated for 20 minutes at room temperature. Afterward, 10 μL of 15% NH_4_HCO_3_ (w/v) was introduced to neutralize the pH of the solvent. The solution was dried down under N_2_ flow, resuspended in 50 μL 40:40:20 (methanol/acetonitrile/water) solvent, and kept at 4°C in an autosampler.

Samples were analyzed using a Q Exactive Plus mass spectrometer coupled to Vanquish UHPLC system (Thermo Fisher Scientific). LC separation was achieved using a XBridge BEH Amide column (2.1 mm × 150 mm, 2.5-μm particle size, 130-Å pore size; Waters, Milford, MA, USA) using a gradient of solvent A (20 mM ammonium acetate + 20 mM ammonium hydroxide in 95:5 water/acetonitrile [pH 9.45]) and solvent B (acetonitrile). Flow rate was 150 μl/min. The gradient was 0 min, 90% B; 2 min, 90% B; 5 min, 50% B; 10 min, 0% B; 13.5 min, 0% B; 15 min, 90% B; 20 min, 90% B. The column temperature was 25°C, and the injection volume was 10 μL. The mass spectrometer parameters are positive ion mode, resolution 140,000 at m/z 200, scan range m/z 290–650, AGC target 3E6, maximum injection time 200 ms. Quantitation of GSH concentrations in samples were achieved by comparing the peak areas of glutathione to those of ^13^C-GSH. Data were analyzed using the MAVEN software [80].

### Competition

To quantify multiple competition replicates at multiple initial strain ratios, we used the coculture system to mimic the lysine-limited environment, especially because similar mutations in coculture and monoculture lysine-limited chemostats meant that the environments were similar. To do so, WY1340 (the purine-requiring/lysine-releasing strain in the RM11 background) was grown to exponential phase overnight in SD + 134 μM adenine, washed 3 times with SD to remove adenine, and starved for 24 hours to deplete vacuolar storage. During this starvation, WY2072/2073 (BFP *met10*-evolved clones) and WY2429 (mCherry *MET10*-evolved clone) were grown overnight in SD + 134 μM methionine to exponential phase and washed 3 times with SD to remove excess methionine and lysine. Both WY2072/3 and WY2429 overproduce and release hypoxanthine which can support the growth of the partner strain WY1340. Next, WY2072/3 and WY2429 were mixed in ratios of 1:100, 1:10, 1:1, and 10:1 to a final OD_600_ of 0.1. This mixture of populations was then added 1:1 with WY1340 to a final OD_600_ of 0.03. This was considered generation 0. Populations were monitored for growth by measuring optical density over time and periodically diluted back to OD_600_ 0.03 (OD_600_ was never greater than 0.45 to ensure no additional metabolites from SD was limiting). The OD_600_ data were used to back-calculate total generations in the experiment. Periodically, 100 μL of the culture was sampled for flow cytometry to track strain ratios. Experiments were performed until the strain ratio stabilized.

### Autophagy assay

Autophagy activities were measured using the GFP-Atg8 cleavage assay [28]. Yeast strains with *ura3* deletion in *lys*^−^ and *lys*^−^*met17*^−^ background were generated via crosses and transformed with GFP-Atg8 plasmid (Addgene 49425; Watertown, MA, USA) to generate the 2 strains used in the autophagy assays—WY2520 (*lys*^−^) and WY2521 (*lys*^−^*orgS*^−^). This plasmid expresses ATG8 with an N-terminal GFP tag under the endogenous promoter in a pRS416 vector with a *URA3* selection marker [81]. For every experiment, WY2520 and WY2521 were streaked on SC-Ura [74] plates from frozen stocks and saturated overnights were grown from single colonies in SC-Ura medium. Cultures of 25–50 mL volume were inoculated from the overnights in SD + 164 μM lysine (WY2520) or SD + 164 μM lysine + 134 μM GSH (WY2521) in conical flasks and grown for 18–20 hours at 30°C to a desirable cell density. In accordance with published protocols, the initial trials aimed at a starting OD of 0.7–1.0 for starvation. However, we observed that high cell densities could result in higher GFP-Atg8 cleavage even in unstarved cells. Thus, in subsequent trials, starvation was initiated at an OD in the range of 0.2–0.6. The cells were pelleted in 50-mL falcon tubes and washed thrice in sterile milliQ water. After the washes, cells were resuspended in the starvation medium of choice (see details below) in factory-clean tubes at an OD of 0.1–0.2. For the conditions in which cells did not arrest growth upon starvation (primarily organosulfur starvation), cultures were periodically diluted to minimize the influence of secondary nutrient limitations caused by high cell densities. For time-course analysis, starvation was carried out in 10-mL cultures in 18-mm tubes, and 2 mL of the culture was withdrawn for analysis at 24, 48, and 72 hours from the initiation of starvation. For assays with a single time-point sampling, starvation was carried out in 3-mL cultures in 13-mm tubes. *lys*^−^ cells were starved for 4 or 8 hours, with comparable results observed for both treatments. *lys*^−^*orgS*^−^ cells were starved for 72 hours after the time-course analysis revealed that the influence of organosulfur starvation was only discernable after 48 hours in the GFP-Atg8 cleavage assay.

Starvation media for different treatments are described here. For *lys*^−^ cells, lysine starvation was carried out in SD medium; nitrogen starvations were carried out in SD-N medium either supplemented with 164 μM lysine (only nitrogen starvation) or lacking it (double starvation for lysine and nitrogen). For *lys*^−^*orgS*^−^ cells, double starvation for lysine and organosulfur was carried out in SD medium, lysine starvation was carried out in SD + 134 μM GSH, and organosulfur starvation was carried out in SD + 164 μM lysine.

Sample preparation was carried out as suggested in [82] with minor modifications. Cells from 2–4 ml of culture were pelleted in a microcentrifuge tube at 5,000 × *g* for 3 minutes. Cell pellets were flash-frozen in liquid nitrogen and stored in −20°C till all samples had been collected for an experiment. For cell lysis, pellets were resuspended in 1 mL of ice-cold 10% trichloroacetic acid and allowed to stand in a cold metal block on ice for 30–40 minutes. Proteins were pelleted at 16,000 × *g* for 1 minute at 4°C. The pellets were resuspended in 1 mL cold acetone by vortexing and bath sonication and pelleted again by centrifugation. The acetone wash was repeated once and pellets were allowed to air-dry for 5 minutes before resuspending in SDS-PAGE sample buffer (0.1 M Tris-HCl [pH 7.5], 2% w/v SDS, 10% v/v glycerol, 20 mM DTT). Based on the OD measured at the time of sample collection, the sample buffer volume was adjusted to attain comparable cells/μL in each sample. Acid-washed glass beads (425–600 μm; Sigma G8771; Sigma-Aldrich, St. Louis, MO, USA) were added to each tube, roughly equivalent to half the sample volume, and the pellet was resuspended by bead beating for 35 seconds. After centrifugation for 20 minutes to allow the foam to settle, the samples were heated in a 95°C metal block for 10 minutes. After a 3-minute centrifugation at 5,000 × *g*, samples were run on a 12.5% acrylamide gel for 50 minutes at a constant current of 30 mA. The bands were transferred onto a 0.2-μm PVDF membrane (Bio-Rad Trans-blot 162–0184) using a wet transfer protocol at a constant voltage of 60 V for 2 hours in the cold room. For immunoblotting, the membrane was incubated overnight at 4°C with an anti-GFP monoclonal primary (JL-8; Clontech 632381; Clontech, Takara Bio, Mountain View, CA, USA) at 1:5,000 dilution, followed by a 45-minute room temperature incubation with a horseradish-peroxidase–conjugated anti-mouse secondary (GE Healthcare Lifesciences [now known as Cytiva]; Marlborough, MA, USA) at 1:10,000 dilution. Antibodies were detected using the SuperSignal West Dura Chemiluminescent Substrate (Thermo Fisher Scientific). The preinstalled Gel Analyzer plugin on ImageJ was used for quantification of bands.

## Supporting information

S1 Fig Decrease in total fluorescence can be used as a proxy for loss of cell viability. Loss of fluorescence represents cell death, as supported by 3 lines of evidence. First, in time-lapse movies, as a cell ruptured, its fluorescence abruptly disappeared (Supplementary Movies 3 and 4 in [27]). Second, regardless of whether live cells were defined as fluorescent-protein–expressing cells, colony-forming cells, or cells with intact membrane and thus capable of excluding the nucleic acid dye ToPro3, we obtained similar quantification of live-cell densities. Specifically, adenine-auxotrophic (A) and lysine-auxotrophic (B) cells expressing fluorescent proteins were grown in adenine-limiting and lysine-limiting chemostats, respectively [30,77]. After cell density had reached a steady state, a sample was taken in which a portion was plated on rich medium, and live-cell density was subsequently quantified from colony counts. Another portion of the sample was analyzed by flow cytometry after being stained with ToPro3, a nucleic acid dye that can only enter cells with compromised membrane integrity. Live-cell density was then quantified either from cells that excluded ToPro or from cells that expressed fluorescent proteins. Each comparison was for an independently run chemostat. We can see that the 3 assays generated similar quantifications of live-cell densities. Third, death rate quantified from the decline in total fluorescence during nutrient starvation was similar to that quantified from the decline in ToPro3-negative live cells [27]. Error bars mark 2 standard deviations. Plotted data are provided in S10 Data. fluo, fluorescent.
(TIF)

S2 Fig Cell viability during nutrient starvation is reduced by activating growth. Bcy1 inhibits the Ras/PKA growth-activating pathway, and thus *bcy1Δ* suffers overactive growth. Compared to *lys*^−^ cells (WY2490) under dual starvation for lysine and glucose, *lys*^−^*bcy1Δ* (WY2527) cells suffered reduced cell viability. Exponentially growing cells were washed and starved for glucose and lysine for 3 h and cultured and imaged in minimal medium without glucose or lysine (Methods, “Fluorescence microscopy”). The initial increase in the fluorescence of *lys*^−^ cells was due to cells becoming brighter. Error bars correspond to 2 standard deviations for 6 replicate wells. Plotted data are provided in S1 Data. Bcy1, bypass of cyclic-AMP requirement protein 1; *lys*^−^; lysine-requiring mutant; PKA, protein kinase A.
(TIF)

S3 Fig *lys*^−^*orgS*^−^ repeatedly rose to detectable frequency when *lys*^−^ cells were limited for lysine. *lys*^−^ cells (WY1335) were either cocultured (crosses) with a lysine-releasing strain (WY1340) or cultured alone in lysine-limited chemostats (circles). Cultures were frozen periodically in 1:1 volume of YPD/20% trehalose in 50 mM NaH_2_PO_4_ (pH 6.0). We revived frozen samples by directly plating samples on YPD or, in case of cocultures, YPD + hygromycin to select against the partner strain. We observed a variety of colony sizes, and since large and small colonies had different percentages of *lys*^−^*orgS*^−^, we quantified both types and calculated the overall *orgS*^−^ in the population (Methods, “Quantifying auxotroph frequency”). Colonies that grew in YPD and in SD + lysine + methionine but failed to grow in SD + lysine were scored as *lys*^−^*orgS*^−^. Different colors indicate independent evolution lines (S11 Data). The fraction of *lys*^−^*orgS*^−^ is much lower in monocultures compared to cocultures. One explanation is that in coculture experiments, the lysine-releasing partner strain also released organosulfurs (Fig 5C). *lys*^−^, lysine-requiring mutant; *met*^−^, methionine-requiring mutant; *orgS*^−^, organosulfur-requiring mutant; SD, synthetic minimal glucose medium; YPD, yeast extract peptone glucose-rich medium.
(TIF)

S4 Fig Rapid accumulation of organosulfurs upon lysine limitation. Ancestral *lys*^−^ cells (WY1335) were grown to exponential phase in SD supplemented with excess lysine, then washed free of lysine and inoculated into replicate lysine-limited chemostats (different symbols) with 8-h doubling time (green) or 4-h doubling time (blue). Periodically, culture supernatants were quickly sampled, filtered, and frozen at −80°C to preserve the redox states of released compounds. Plotted data are provided in S12 Data. (A) Population dynamics of live (fluorescent) and dead (nonfluorescent) cells in chemostats, as quantified by flow cytometry (Methods, “Flow cytometry”). (B) Organosulfurs in supernatants were measured as “methionine equivalents” by comparing growth rates of a *met17*^−^ tester strain (WY2035) in supernatants fortified by SD versus in SD supplemented with known concentrations of methionine (standard curve in inset). Since the growth rate of tester cells can be affected by factors other than organosulfurs (e.g., pH), the organosulfur niche estimated by a rate-based bioassay can differ from that estimated from a turbidity-based bioassay (e.g., Fig 5B). Nevertheless, this assay motivated the LC–MS experiments that identified GSH as one of the released organosulfurs (Fig 4A). (C) The release rate of organosulfurs is higher in 8-h doubling chemostats compared to 4-h doubling chemostats. Release rates were calculated from the first 24 h from independent chemostats run in 2 experiments. If organosulfurs are the major factor affecting *met17* growth rate, then the organosulfur release rate by *lys*^−^ cells in 8-h chemostats is significantly higher than that in 4-h chemostats by *t* test (2-tailed, equal variance). Expt, experiment; LC–MS, liquid chromatography–mass spectrometry; *lys*^−^, lysine-requiring mutant; Met, methionine; Met equiv; methionine equivalent; *orgS*, organosulfur; SD, synthetic minimal glucose medium.
(TIF)

S5 Fig Turbidity-based bioassays of organosulfurs. (A) The sensitivity of turbidity (OD_600_) measurements in a microtiter plate. Purple dotted line marks the lower bound of turbidity reading that we accepted as valid data and is also plotted in B–D. (B) The turbidity of a *gsh1*^−^ strain (WY2509) increases linearly with [GSH] and [GS-SG] within a range but does not increase when supplemented with methionine. Since reduction of one GS-SG molecule generates 2 GSH molecules, culture final turbidity in GS-SG should be twice as much as in equimolar GSH. This is indeed observed. (C, D) *met10*^−^ (WY1604) can use methionine, GSH, and oxidized glutathione (GS-SG). In A–C, error bars represent 2 standard deviations from 3 replicates. (E) *gsh1*^−^ and *met10*^−^ grew to similar levels in supernatants of *lys*^−^ cells grown in lysine-limited chemostats. Error bars are 2 standard deviations from 6 or more data points. All plotted data can be found in S13 Data. GSH, reduced glutathione; GS-SG, glutathione disulfide; *gsh1*^−^, glutathione gene 1 mutant; *lys*^−^, lysine-requiring mutant; *met*^−^, methionine-requiring mutant; OD, optical density.
(TIF)

S6 Fig GSH is likely released by live ancestral and evolved *lys2*^−^ cells. Ancestral (circles; WY1335) and evolved (crosses; WY2429) *lys*^−^ cells were cultured in lysine-limited chemostats (doubling time 8 h). Evolved cells contain an *ecm21* mutation and chromosome 14 duplication, and thus exhibit improved affinity for lysine. Intracellular metabolites were extracted from cells to quantify fmole GSH/cell (brown). We quantified dead-cell density and the concentrations of GSH in culture supernatants. We then calculated the theoretical amount that would need to be inside an average cell for cell lysis alone to explain the supernatant concentrations (purple). Since the theoretical amount was higher than the actual amount in all experiments (note the logarithm scale), GSH is likely released by live cells. GSH was quantified using HPLC, and dead-cell density was quantified using flow cytometry (Methods). Here, each column corresponds to an experiment (S14 Data). Experiment-to-experiment variations exist, but the trend is clear across experiments. Anc, ancestral *lys*^−^ strain; *ECM21*, extracellular mutant gene 21; Evo, evolved *lys*^−^ strain; GSH, reduced glutathione; HPLC, High-Performance Liquid Chromatography; *lys*^−^, lysine-requiring mutant.
(TIF)

S7 Fig Lysine limitation increases GSH release rate. (A–C) Ancestral *lys2*^−^ cells (WY1335) were cultured in excess lysine (turbidostats at 1.5-h doubling time; magenta) or limited lysine (lysine-limited chemostats at 8-h doubling time; green). (A) Live and dead population densities were quantified using flow cytometry (Methods, “Flow cytometry”), and (B) supernatant GSH concentrations were quantified using a fluorescence-based HPLC assay [79] (Methods, “HPLC”). Different symbols represent independent experiments. The dotted line in B marks the sensitivity of the HPLC assay. (C) GSH release rate was higher during lysine limitation. To calculate the release rate of GSH per live cell in chemostats, we used a previously described method [30]. Specifically, the steady-state concentration of glutathione in a chemostat (B) was divided by the live-cell density (A) and then multiplied by dilution rate (/hour). *P*-value was derived from a one-tailed *t* test with equal variance (as per F test). Here, we used HPLC to measure GSH instead of bioassay to measure total organosulfurs because the latter assay was much less sensitive. (D) GSH release rates were comparable between the ancestral *lys2*^−^ (circles) and an evolved *lys2*^−^ clone (crosses; WY2429, which contains an *ecm21* mutation and chromosome 14 duplication and thus exhibits improved affinity for lysine). Here, cells were grown in lysine-limited chemostats (8-h doubling). In (C) and (D), different colors correspond to experiments done on different days, and each symbol represents an independent culture. All plotted data are available in S3 Data. Anc, ancestral *lys*^−^ strain; ECM21, extracellular mutant gene 21; Evo, an evolved *lys*^−^ strain; GSH, reduced glutathione; HPLC, High-Performance Liquid Chromatography; *lys*^−^, lysine-requiring mutant; T_2_, doubling time.
(TIF)

S8 Fig Live adenine-limited *ade*^−^ cells release organosulfurs. *ade*^−^ cells (WY1340; WY1598) were grown in adenine-limited chemostats at 8-hour doubling time. After cultures had reached steady-state cell density (approximately 71–73 hours), a sample was taken to assay for total cell density, live-cell density, and dead-cell density using flow cytometry. Simultaneously, another sample was filtered to harvest supernatant, and a third sample was taken to harvest cells to make extracts. Total organosulfurs in supernatants (brown) and cell extracts were measured via the *met10*-based bioassay. From total organosulfur in extracts and the total number of cells harvested, we calculated fmole organosulfurs per cell. We inferred the contribution to total release by dead cells by multiplying dead-cell density with fmole organosulfur/cell. Six independent experiments are plotted (S5 Data). *ade*^−^, adenine-requiring mutant; GSH equiv., reduced glutathione equivalent; *met*^−^, methionine-requiring mutant.
(TIF)

S9 Fig Adenine-limited *ade*^−^ cells display aspects of nutrient–growth dysregulation. Multiple lines of evidence suggest that adenine-limited *ade*^−^ cells suffer nutrient–growth dysregulation. In our previous work, we found that in very low concentrations (approximately 0.1–0.2 μM) of hypoxanthine (which can be converted to adenine by cells), *ade*^−^ cells initially divided but then died at a faster rate than during adenine starvation, consistent with nutrient–growth dysregulation during adenine limitation (Fig 3C in [27]). Here, exponentially growing *ade*^−^ cells (WY1340) were washed in sterile water and cultured in minimal medium lacking adenine, either with glucose (“+ carbon,” red) or without glucose (“− carbon,” black) for 24 hours before imaging in the same medium. For the rapamycin sample (“+ carbon + rapamycin,” blue), rapamycin was added at the beginning of imaging. After an initial long lag, *ade*^−^ cells died (red). Similar to *lys*^−^ cells, cell death during adenine starvation was mitigated by removing glucose (compare red with black). Unlike *lys*^−^ cells, cell death was not inhibited by rapamycin (compare red with blue). This suggests that during purine starvation, although TORC1 is already inactivated, other pathways (such as Ras/PKA) are still active, contributing to nutrient–growth dysregulation. Error bars correspond to 2 standard deviations from 6 replicate wells. All data are available in S15 Data. *ade*^−^, adenine-requiring mutant; *lys*^−^, lysine-requiring mutant; PKA, protein kinase A; TORC1, target of rapamycin 1
(TIF)

S10 Fig *lys*^−^*orgS*^−^ survives lysine limitation better than *lys*^−^ if organosulfur is also limited. *lys*^−^ (WY2429, blue), *lys*^−^*orgS*^−^ (WY1604, orange), and *lys*^−^*orgS*^−^*atg5*^−^ (WY2370, green) were grown to exponential phase in SD supplemented with excess lysine (164 μM) and excess GSH (134 μM). These cells were washed and starved in SD without lysine or GSH for 5 hours prior to imaging in indicated conditions with 1 μM rapamycin (crosses) or without rapamycin (circles). Total fluorescence normalized against time zero are plotted. All plotted data can be found in S8 Data. (A) When GSH was limited, *lys*^−^*orgS*^−^ survived better than *lys*^−^ (orange circles above blue circles). TORC1 inhibition by rapamycin improved the survival of both *lys*^−^*orgS*^−^ and *lys*^−^ to comparable levels (orange and blue crosses). *lys*^−^*orgS*^−^*atg5*^−^ survived poorly even when TORC1 was shut down (green crosses). (B) At high GSH, both *lys*^−^*orgS*^−^ and *lys*^−^ survived poorly (orange circles trending down at a similar slope as blue circles), and this poor viability was rescued by rapamycin (blue and orange crosses). *lys*^−^*orgS*^−^*atg5*^−^ survived poorly in the presence or absence of rapamycin (green). The approximately 2-fold initial increase in the top curves was due to cell swelling [27]. Error bars represent 2 standard deviations from 4 positions in a well. *atg5*, autophagy-related gene 5 mutant; GSH, reduced glutathione; *lys*^−^, lysine-requiring mutant; rap, rapamycin; *orgS*^−^, organosulfur-requiring mutant; SD, synthetic minimal glucose medium; TORC1, target of rapamycin complex 1
(TIF)

S11 Fig Comparing *lys*^−^*orgS*^−^ and *lys*^−^ cells in various concentrations of GSH and lysine. *lys*^−^ (WY2429, blue) and *lys*^−^*orgS*^−^ (WY1604, orange) cells were grown in SD + excess lysine (164 μM) and excess GSH (134 μM) to exponential phase. These cells were washed and starved in SD for 24 hours and imaged in various concentrations of GSH and lysine. During the growth phase, *lys*^−^ grew faster than *lys*^−^*orgS*^−^ in most cases. After lysine was exhausted, *lys*^−^*orgS*^−^ survived better than *lys*^−^. Note that in the microscopy assay, the minimal medium did not contain GSX or other excreted compounds found in culture supernatants, and thus, results are not directly comparable to competition experiments. Regardless, and consistent with the competition experiment, *lys*^−^*orgS*^−^ cells grew faster than *lys*^−^ cells under certain conditions. In this experiment, at 2 μM GSH and 0.1 μM lysine, the maximal growth rate achieved by *lys*^−^*orgS*^−^ was 0.153 ± 0.009/hour (mean ± 2 standard error of mean), greater than the 0.133 ± 0.004/hour achieved by *lys*^−^. Fluorescence intensities of various time points are normalized against that at time zero. Plotted data can be found in S17 Data. GSH, reduced glutathione; GSX, glutathione-S-conjugate; *lys*^−^, lysine-requiring mutant; *orgS*^−^, organosulfur-requiring mutant; SD, synthetic minimal glucose medium.
(TIF)

S12 Fig Autophagy activity is higher when *lys*^−^*orgS*^−^ cells are starved of both organosulfurs and lysine compared to starved of lysine alone. The ideal comparison of autophagy between *lys*^−^ and *lys*^−^*orgS*^−^ cells has multiple technical difficulties. Cells should ideally be cultured in an environment that mimics the original evolutionary environment (i.e., in low concentrations of lysine and organosulfurs). This means that *lys*^−^*orgS*^−^ cells would need to be cultured in a chemostat dual-limited for sulfur and lysine. However, the theory of chemostat is based on single-nutrient limitation [35], and ensuring dual limitation is nontrivial. Thus, we assayed autophagy in batch starvation cultures. Unfortunately in batch starvation cultures, it can be difficult to compare autophagy activities between different genotypes; e.g., the kinetics of autophagy and death differed drastically between *lys*^−^ and *lys*^−^*orgS*^−^. When supplied with excess lysine and no organosulfurs, *lys*^−^*orgS*^−^ cells continued to divide for multiple rounds using internal organosulfur storage, and autophagy induction was very slow. In contrast, lysine-starved *lys*^−^ cells died quickly. Thus, a comparison between the 2 genotypes is difficult. For these reasons, we compared autophagy activities in batch cultures of a single genotype (*lys*^−^*orgS*^−^; WY2520) under different starvation conditions. Using the GFP-Atg8 cleavage assay, we observed a moderate increase in autophagy when *lys*^−^*orgS*^−^ cells were starved for both lysine and organosulfurs as opposed to only lysine starvation. Plotted data are available in S16 Data. (A) Time course of autophagy induction during lysine starvation (orange), organosulfur starvation (purple), and dual starvation (green). (B) Results from 4 trials (the fourth trial is identical to A). Autophagy of exponential cultures (first bar in each set) and of singly or doubly starved cultures at 72 h (second to fourth bars in each set) are plotted. (C) Autophagy activity was generally higher in cells starved for both lysine and organosulfurs than in cells starved for lysine only. A one-sample *t* test against the null hypothesis of identical autophagy activities (ratio = 1) gives *P* = 0.03, although given the few data points, we cannot be certain that the data satisfy the *t* test assumption of normal distribution. Atg8, autophagy-related protein 8; GFP, green fluorescent protein; *lys*^−^, lysine-requiring mutant; *orgS*^−^, organosulfur-requiring mutant.
(TIF)

S13 Fig Negative-frequency–dependent selection for *lys*^−^*orgS*^−^. BFP-tagged *lys*^−^*orgS*^−^ (WY2072 or WY2073) and mCherry-tagged *lys*^−^ (WY2039 or WY2045) were placed in competition in a lysine-limited environment by coculturing with a lysine-releasing strain (WY1340). Strain ratios over time were measured by flow cytometry (Fig 6C). For each trajectory, we computed the slope of *ln*(*lys*^−^*orgS*^−^/*lys*^−^) over 3 consecutive time points and chose the steepest slope. Since our time unit was generation, we divided this slope (/generation) by *ln*2/generation and obtained a dimensionless number representing the relative fitness difference between *lys*^−^*orgS*^−^ and *lys*^−^. We then plotted the relative fitness difference against the fraction of *lys*^−^*orgS*^−^ at the beginning of the time window used to calculate the steepest slope. Dotted line marks equal fitness between the 2 strains. The fitness advantage of *lys*^−^*orgS*^−^ over *lys*^−^ decreases as the fraction of *lys*^−^*orgS*^−^ increases (i.e., negative-frequency–dependent). Plotted data are provided in S9 Data. BFP, blue fluorescent protein; *lys*^−^, lysine-requiring mutant; *orgS*^−^, organosulfur-requiring mutant.
(TIF)

S14 Fig Continuous culturing device. The continuous culturing device (A) consists of 6 channels that can independently operate as a chemostat or a turbidostat. Each channel (B) consists of a culturing vessel (center), a magnetic stirrer, an LED-phototransistor optical detector for OD measurement, a computer-activated pump, a media reservoir (left), and a scale for measuring media reservoir (and flow rate). A LabView program running on the CPU uses data from the scale or the optical detector to control the pump, which maintains a constant average OD in the turbidostat mode, or a constant average flow rate in the chemostat mode. Each vessel (C) consists of a Pyrex test tube modified by adding a waste outlet of adequate diameter and slope to ensure a reliable flow of waste driven by gravity. The vessel’s rubber stopper had a sampling port consisting of a needle that can be raised and lowered through a segment of PharMed tubing, which was held in place by glass tubing inserted into the stopper. The tightness of the seal between the sampling needle and PharMed tubing can be adjusted using a zip-tie, allowing easy motion of the needle while maintaining its position when stationary. The 6 vessels, stirrers, and photodetectors are held in position in a frame cut from an aluminum bar. The signal from each phototransistor was converted to a voltage using an op-amp current to voltage converter (box in B). (D) Constant flow rates in chemostats. An example is shown. (E) Culturing vessel volume (depth approximately 125 mm) averages 43 ml. Individual culturing vessel volume was used for converting doubling time to flow rate and was measured to approximately 0.5-ml resolution (limited by minimum outflow drop size; error bar). Data for D–E can be found in S18 Data. CPU, Central Processing Unit; DAQ, Data Acquisition system; LED, light-emitting diode; OD, optical density; PTFE, Polytetrafluoroethylene.
(TIF)

S1 Text Additional perspectives on nutrient–growth regulation. We discuss how our model of nutrient–growth regulation (Fig 1) may be reconciled with seemingly conflicting biochemistry studies on the response of TORC1 to unnatural limitation [83,84]. TORC1, target of rapamycin complex 1.
(DOCX)

S1 Table Strains used in this study. (XLSX)

S2 Table Mutations in evolved clones. All clones with the alias “ACI” are from chemostat evolution experiment. All other clones are from coculture evolution experiments, a fraction of which (i.e., strains with alias “CT”) are from an earlier study [31]. Gray shading: auxotrophic strain. For WY2467, we identified it as *orgS*− from growth patterns. However, its genetic basis is unclear since among identified mutations, none is known to affect organosulfur biosynthesis. Matched color shading: mutation adaptive to lysine limitation shared between a *lys*^−^ clone and a *lys*^−^*orgS*^−^ clone from the same culture. *lys*^−^, lysine-requiring mutant; *orgS*^−^, organosulfur-requiring mutant.
(XLSX)

S1 Data Data plotted in Fig 2 and S2 Fig. (XLSX)

S2 Data Data plotted in Fig 4A. (XLSX)

S3 Data Data plotted in S7 Fig. (XLSX)

S4 Data Data plotted in Fig 4B. (XLSX)

S5 Data Data plotted in Figs 4C and 5C and S8 Fig. (XLSX)

S6 Data Data plotted in Fig 5A and 5B. (XLSX)

S7 Data Data plotted in Fig 6A. (XLSX)

S8 Data Data plotted in Fig 6B and S10 Fig. (XLSX)

S9 Data Data plotted in Fig 6C and S13 Fig. (XLSX)

S10 Data Data plotted in S1 Fig. (XLSX)

S11 Data Data plotted in S3 Fig. (XLSX)

S12 Data Data plotted in S4 Fig. (XLSX)

S13 Data Data plotted in S5 Fig. (XLSX)

S14 Data Data plotted in S6 Fig. (XLSX)

S15 Data Data plotted in S9 Fig. (XLSX)

S16 Data Data plotted in S12 Fig. (XLSX)

S17 Data Data plotted in S11 Fig. (XLSX)

S18 Data Data plotted in S14 Fig. (XLSX)

S1 Raw Images Original western blots for data in Fig 2B and S12 Fig. (PDF)

## Abbreviations

*ade*^−^: adenine-requiring mutant
*atg5*: autophagy-related gene 5 mutant
Atg8: autophagy-related protein 8
Bcy1: bypass of cyclic-AMP requirement protein 1
Bpt1: bile pigment transporter protein 1
CoA: Coenzyme A
*DISOMY14*: duplication of chromosome 14
*DOA4*: degradation of alpha gene 4
*ECM21*: extracellular mutant gene 21
Gex: glutathione exchanger
GFP: green fluorescent protein
*gln1*: glutamine metabolism gene 1 mutant
GSH: reduced glutathione
*gsh1*: glutathione gene 1 mutant
GSX: glutathione S-conjugate
GS-SG: glutathione disulfide
Gxa1: glutathione export ABC protein 1
HPLC: High-Performance Liquid Chromatography
LC–MS: liquid chromatography–mass spectrometry
*leu*^−^: leucine-requiring mutant
*LYP1*: lysine-specific permease gene 1
*lys*^−^: lysine-requiring mutant
*met*^−^: methionine-requiring mutant
Opt1: oligo peptide transporter protein 1
orgS: organosulfur
PKA: protein kinase A
*PPM1*: protein phosphatase methyltransferase gene 1
*RSP5*: reverses spt-phenotype gene 5
*SCH9*: gene encoding a serine/threonine protein kinase
SD: synthetic minimal glucose medium
TCA: tricarboxylic acid
TCEP: tris(2-carboxyethyl)phosphine
TORC1: target of rapamycin complex 1
Ycf1: yeast cadmium factor protein 1
