# Supplementary figures and images for: Metabolic excretion associated with nutrient–growth dysregulation promotes the rapid evolution of an overt metabolic defect
Source: PLoS Biol. 2020 Aug 24;18(8):e3000757. doi: 10.1371/journal.pbio.3000757 (PMC7470746; doi:10.1371/journal.pbio.3000757)

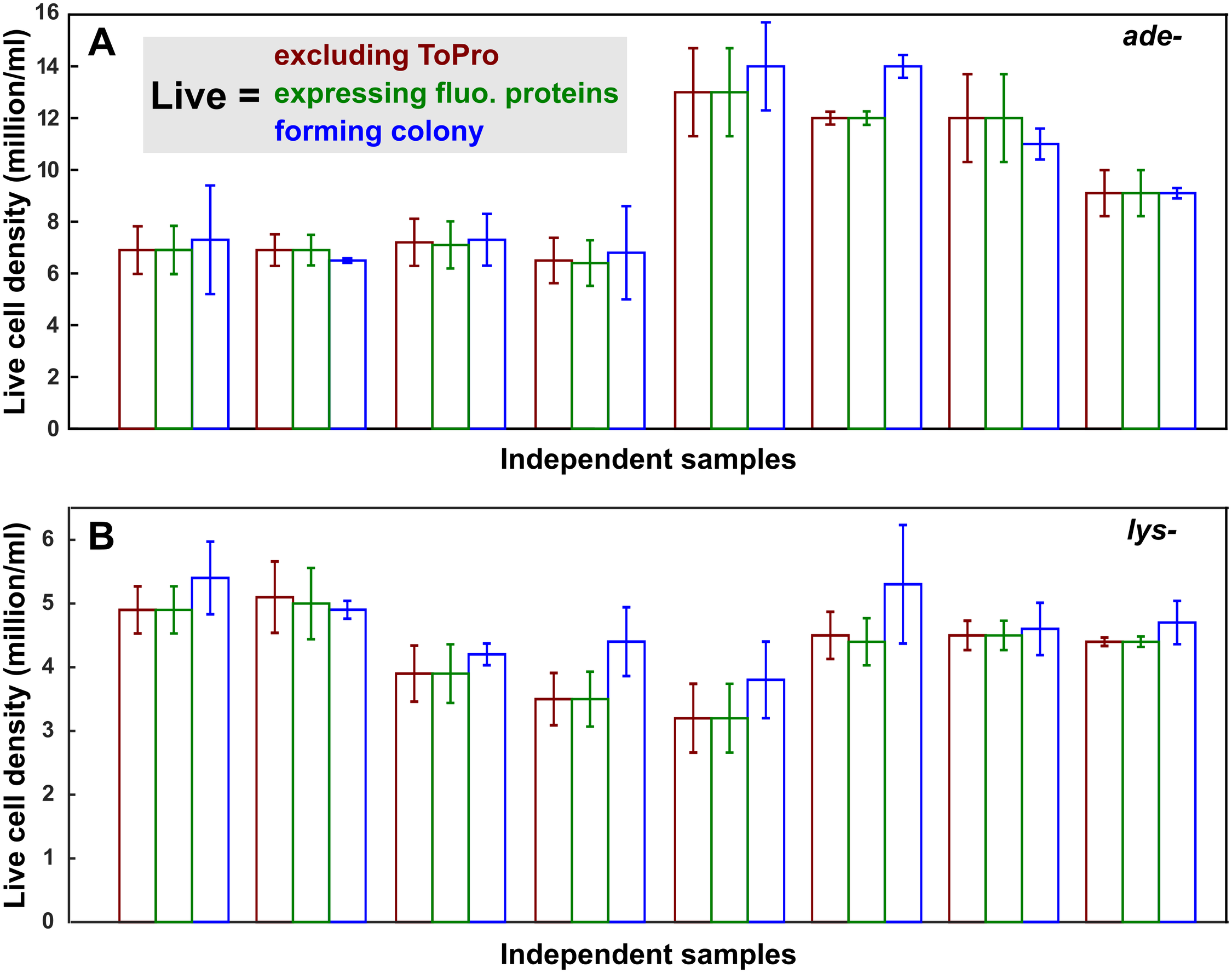

Supplement: S1 Fig — Loss of fluorescence represents cell death, as supported by 3 lines of evidence. First, in time-lapse movies, as a cell ruptured, its fluorescence abruptly disappeared (Supplementary Movies 3 and 4 in [27]). Second, regardless of whether live cells were defined as fluorescent-protein–expressing cells, colony-forming cells, or cells with intact membrane and thus capable of excluding the nucleic acid dye ToPro3, we obtained similar quantification of live-cell densities. Specifically, adenine-auxotrophic (A) and lysine-auxotrophic (B) cells expressing fluorescent proteins were grown in adenine-limiting and lysine-limiting chemostats, respectively [30,77]. After cell density had reached a steady state, a sample was taken in which a portion was plated on rich medium, and live-cell density was subsequently quantified from colony counts. Another portion of the sample was analyzed by flow cytometry after being stained with ToPro3, a nucleic acid dye that can only enter cells with compromised membrane integrity. Live-cell density was then quantified either from cells that excluded ToPro or from cells that expressed fluorescent proteins. Each comparison was for an independently run chemostat. We can see that the 3 assays generated similar quantifications of live-cell densities. Third, death rate quantified from the decline in total fluorescence during nutrient starvation was similar to that quantified from the decline in ToPro3-negative live cells [27]. Error bars mark 2 standard deviations. Plotted data are provided in S10 Data. fluo, fluorescent. (TIF) [file pbio.3000757.s001.tif]

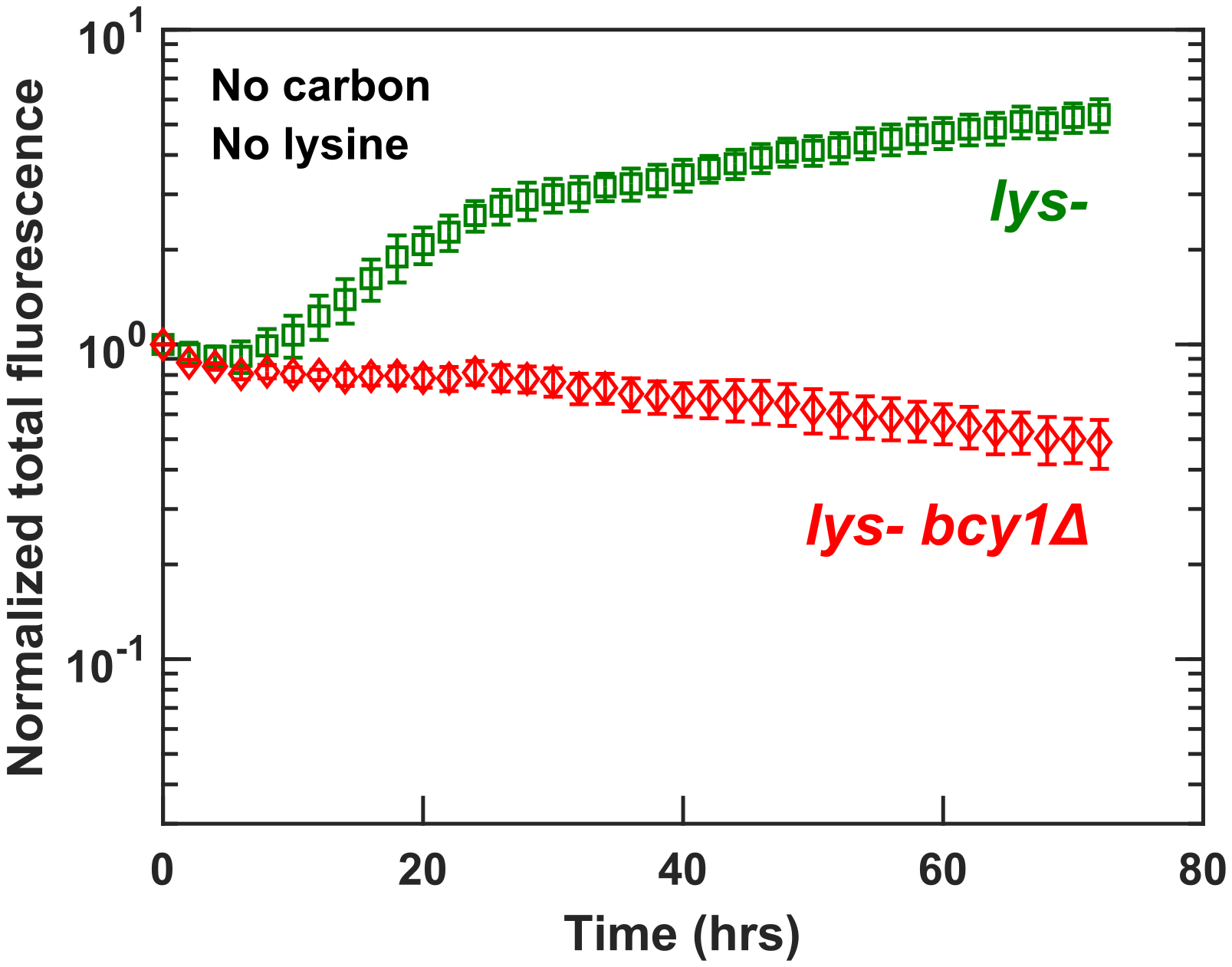

Supplement: S2 Fig — Bcy1 inhibits the Ras/PKA growth-activating pathway, and thus bcy1Δ suffers overactive growth. Compared to lys− cells (WY2490) under dual starvation for lysine and glucose, lys−bcy1Δ (WY2527) cells suffered reduced cell viability. Exponentially growing cells were washed and starved for glucose and lysine for 3 h and cultured and imaged in minimal medium without glucose or lysine (Methods, “Fluorescence microscopy”). The initial increase in the fluorescence of lys− cells was due to cells becoming brighter. Error bars correspond to 2 standard deviations for 6 replicate wells. Plotted data are provided in S1 Data. Bcy1, bypass of cyclic-AMP requirement protein 1; lys−; lysine-requiring mutant; PKA, protein kinase A. (TIF) [file pbio.3000757.s002.tif]

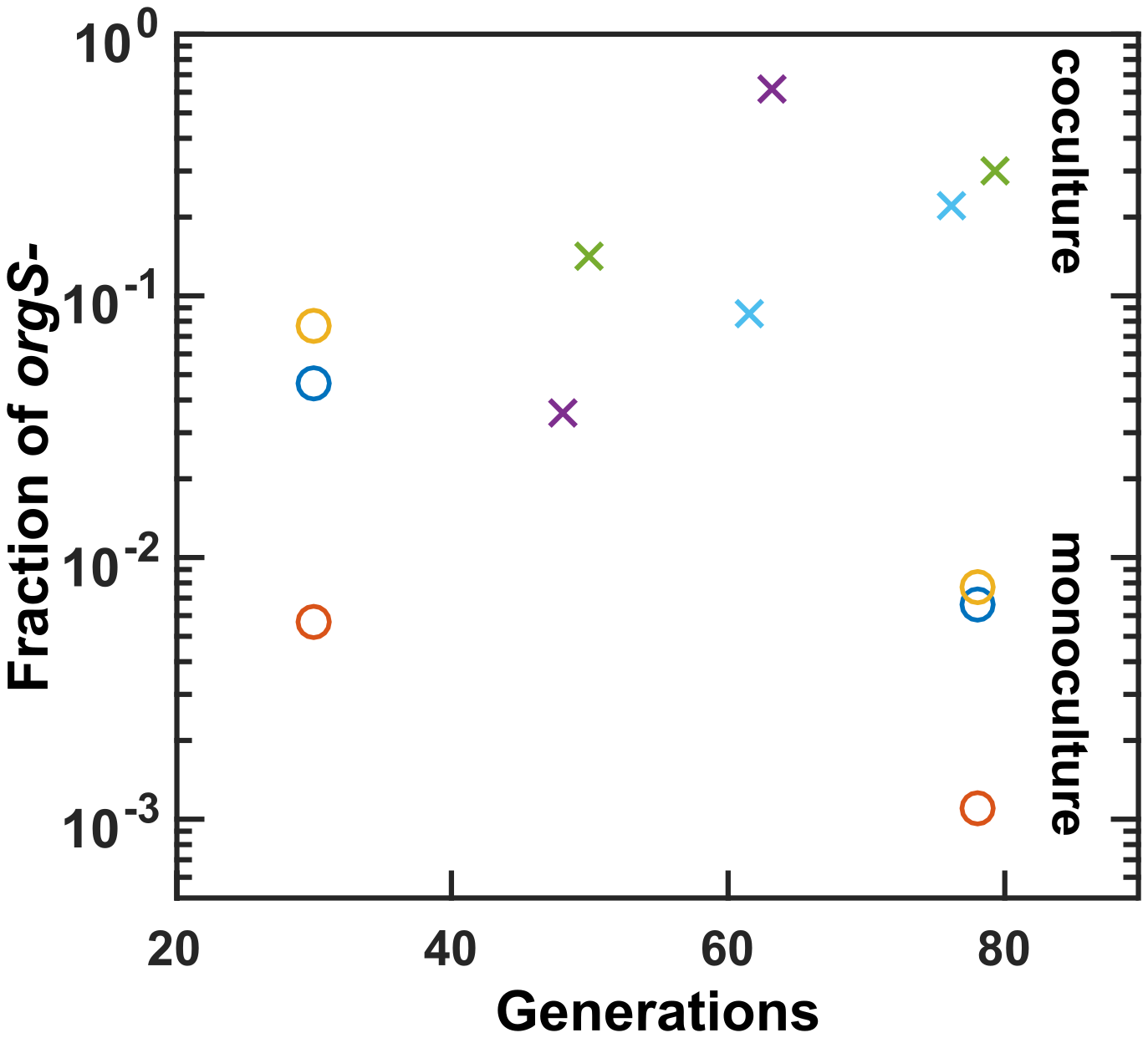

Supplement: S3 Fig — lys− cells (WY1335) were either cocultured (crosses) with a lysine-releasing strain (WY1340) or cultured alone in lysine-limited chemostats (circles). Cultures were frozen periodically in 1:1 volume of YPD/20% trehalose in 50 mM NaH2PO4 (pH 6.0). We revived frozen samples by directly plating samples on YPD or, in case of cocultures, YPD + hygromycin to select against the partner strain. We observed a variety of colony sizes, and since large and small colonies had different percentages of lys−orgS−, we quantified both types and calculated the overall orgS− in the population (Methods, “Quantifying auxotroph frequency”). Colonies that grew in YPD and in SD + lysine + methionine but failed to grow in SD + lysine were scored as lys−orgS−. Different colors indicate independent evolution lines (S11 Data). The fraction of lys−orgS− is much lower in monocultures compared to cocultures. One explanation is that in coculture experiments, the lysine-releasing partner strain also released organosulfurs (Fig 5C). lys−, lysine-requiring mutant; met−, methionine-requiring mutant; orgS−, organosulfur-requiring mutant; SD, synthetic minimal glucose medium; YPD, yeast extract peptone glucose-rich medium. (TIF) [file pbio.3000757.s003.tif]

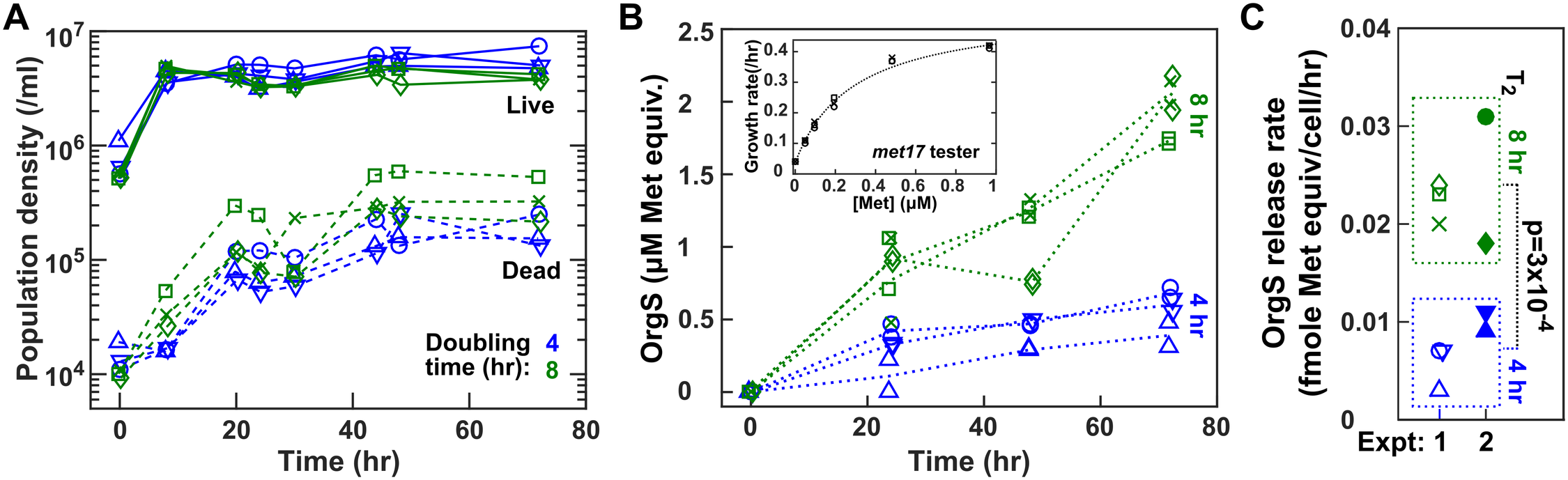

Supplement: S4 Fig — Ancestral lys− cells (WY1335) were grown to exponential phase in SD supplemented with excess lysine, then washed free of lysine and inoculated into replicate lysine-limited chemostats (different symbols) with 8-h doubling time (green) or 4-h doubling time (blue). Periodically, culture supernatants were quickly sampled, filtered, and frozen at −80°C to preserve the redox states of released compounds. Plotted data are provided in S12 Data. (A) Population dynamics of live (fluorescent) and dead (nonfluorescent) cells in chemostats, as quantified by flow cytometry (Methods, “Flow cytometry”). (B) Organosulfurs in supernatants were measured as “methionine equivalents” by comparing growth rates of a met17− tester strain (WY2035) in supernatants fortified by SD versus in SD supplemented with known concentrations of methionine (standard curve in inset). Since the growth rate of tester cells can be affected by factors other than organosulfurs (e.g., pH), the organosulfur niche estimated by a rate-based bioassay can differ from that estimated from a turbidity-based bioassay (e.g., Fig 5B). Nevertheless, this assay motivated the LC–MS experiments that identified GSH as one of the released organosulfurs (Fig 4A). (C) The release rate of organosulfurs is higher in 8-h doubling chemostats compared to 4-h doubling chemostats. Release rates were calculated from the first 24 h from independent chemostats run in 2 experiments. If organosulfurs are the major factor affecting met17 growth rate, then the organosulfur release rate by lys− cells in 8-h chemostats is significantly higher than that in 4-h chemostats by t test (2-tailed, equal variance). Expt, experiment; LC–MS, liquid chromatography–mass spectrometry; lys−, lysine-requiring mutant; Met, methionine; Met equiv; methionine equivalent; orgS, organosulfur; SD, synthetic minimal glucose medium. (TIF) [file pbio.3000757.s004.tif]

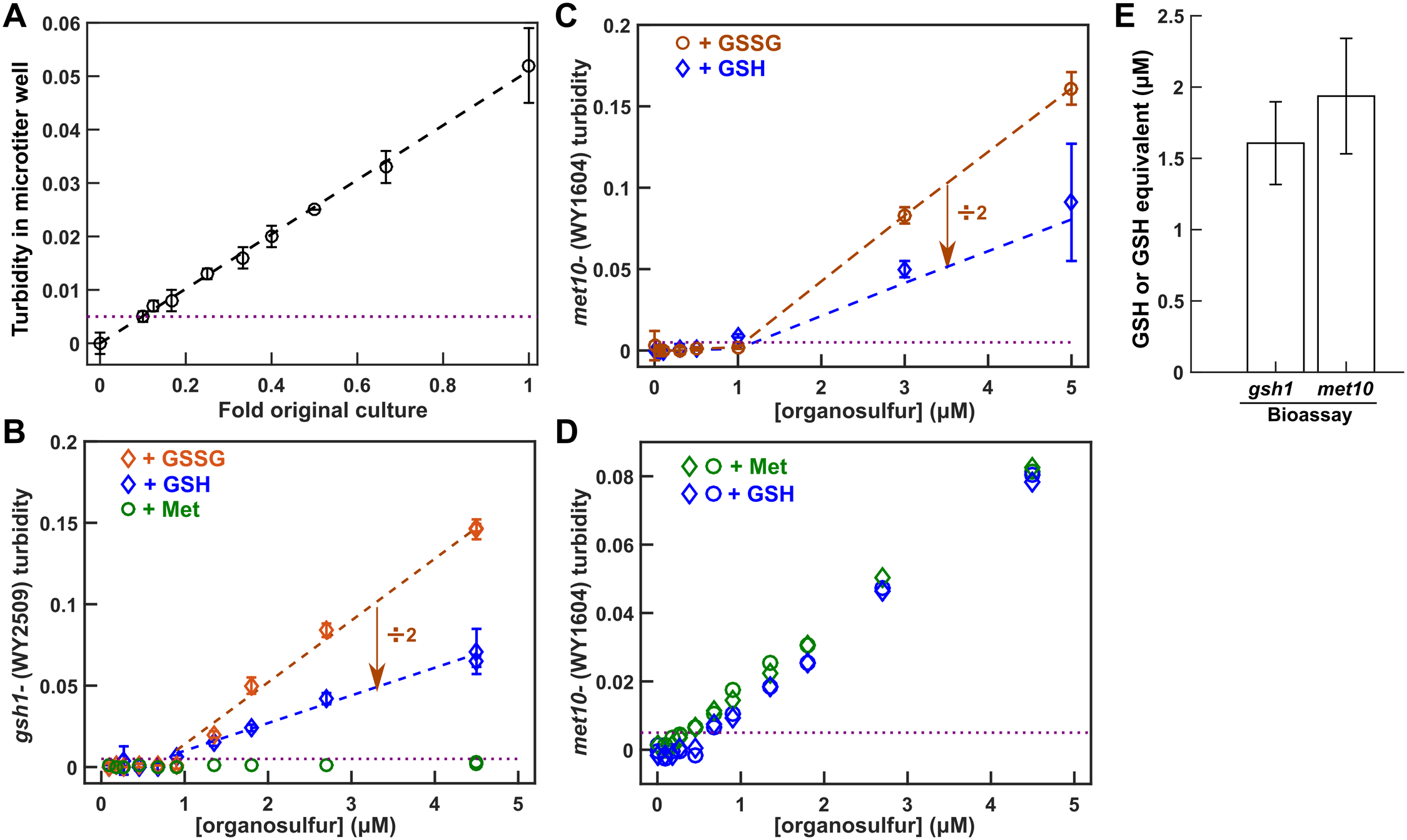

Supplement: S5 Fig — (A) The sensitivity of turbidity (OD600) measurements in a microtiter plate. Purple dotted line marks the lower bound of turbidity reading that we accepted as valid data and is also plotted in B–D. (B) The turbidity of a gsh1− strain (WY2509) increases linearly with [GSH] and [GS-SG] within a range but does not increase when supplemented with methionine. Since reduction of one GS-SG molecule generates 2 GSH molecules, culture final turbidity in GS-SG should be twice as much as in equimolar GSH. This is indeed observed. (C, D) met10− (WY1604) can use methionine, GSH, and oxidized glutathione (GS-SG). In A–C, error bars represent 2 standard deviations from 3 replicates. (E) gsh1− and met10− grew to similar levels in supernatants of lys− cells grown in lysine-limited chemostats. Error bars are 2 standard deviations from 6 or more data points. All plotted data can be found in S13 Data. GSH, reduced glutathione; GS-SG, glutathione disulfide; gsh1−, glutathione gene 1 mutant; lys−, lysine-requiring mutant; met−, methionine-requiring mutant; OD, optical density. (TIF) [file pbio.3000757.s005.tif]

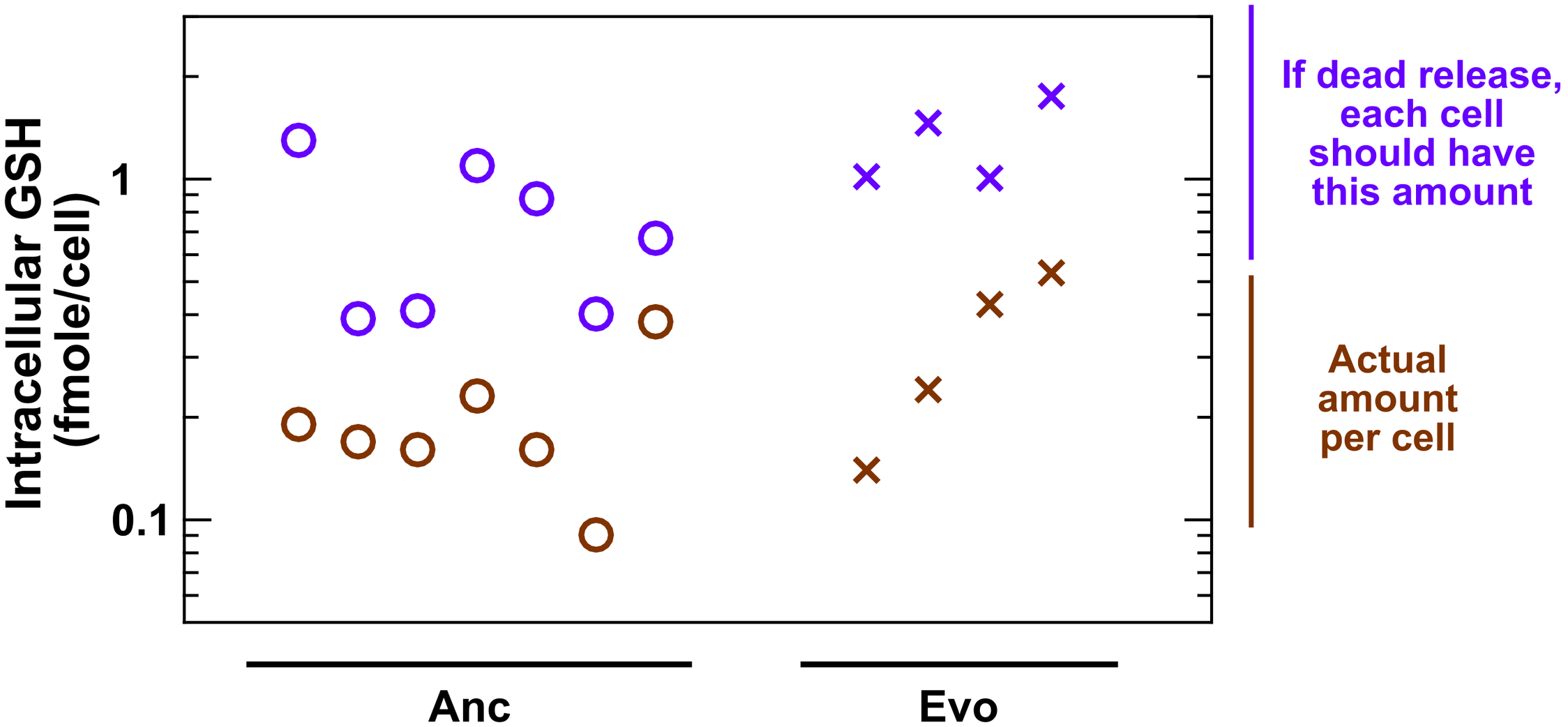

Supplement: S6 Fig — Ancestral (circles; WY1335) and evolved (crosses; WY2429) lys− cells were cultured in lysine-limited chemostats (doubling time 8 h). Evolved cells contain an ecm21 mutation and chromosome 14 duplication, and thus exhibit improved affinity for lysine. Intracellular metabolites were extracted from cells to quantify fmole GSH/cell (brown). We quantified dead-cell density and the concentrations of GSH in culture supernatants. We then calculated the theoretical amount that would need to be inside an average cell for cell lysis alone to explain the supernatant concentrations (purple). Since the theoretical amount was higher than the actual amount in all experiments (note the logarithm scale), GSH is likely released by live cells. GSH was quantified using HPLC, and dead-cell density was quantified using flow cytometry (Methods). Here, each column corresponds to an experiment (S14 Data). Experiment-to-experiment variations exist, but the trend is clear across experiments. Anc, ancestral lys− strain; ECM21, extracellular mutant gene 21; Evo, evolved lys− strain; GSH, reduced glutathione; HPLC, High-Performance Liquid Chromatography; lys−, lysine-requiring mutant. (TIF) [file pbio.3000757.s006.tif]

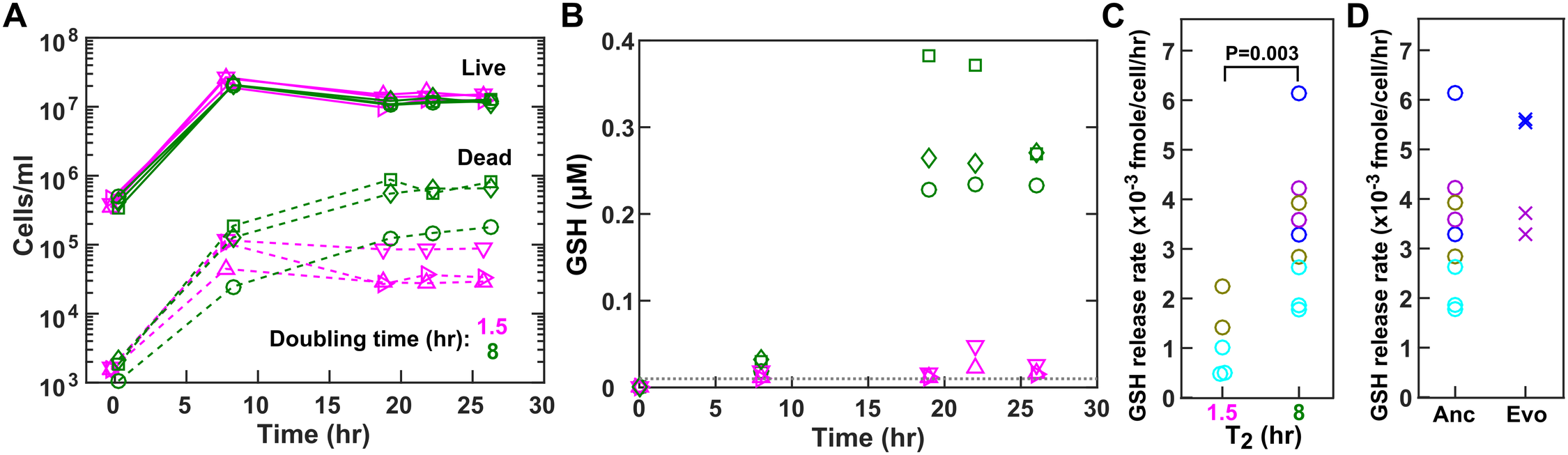

Supplement: S7 Fig — (A–C) Ancestral lys2− cells (WY1335) were cultured in excess lysine (turbidostats at 1.5-h doubling time; magenta) or limited lysine (lysine-limited chemostats at 8-h doubling time; green). (A) Live and dead population densities were quantified using flow cytometry (Methods, “Flow cytometry”), and (B) supernatant GSH concentrations were quantified using a fluorescence-based HPLC assay [79] (Methods, “HPLC”). Different symbols represent independent experiments. The dotted line in B marks the sensitivity of the HPLC assay. (C) GSH release rate was higher during lysine limitation. To calculate the release rate of GSH per live cell in chemostats, we used a previously described method [30]. Specifically, the steady-state concentration of glutathione in a chemostat (B) was divided by the live-cell density (A) and then multiplied by dilution rate (/hour). P-value was derived from a one-tailed t test with equal variance (as per F test). Here, we used HPLC to measure GSH instead of bioassay to measure total organosulfurs because the latter assay was much less sensitive. (D) GSH release rates were comparable between the ancestral lys2− (circles) and an evolved lys2− clone (crosses; WY2429, which contains an ecm21 mutation and chromosome 14 duplication and thus exhibits improved affinity for lysine). Here, cells were grown in lysine-limited chemostats (8-h doubling). In (C) and (D), different colors correspond to experiments done on different days, and each symbol represents an independent culture. All plotted data are available in S3 Data. Anc, ancestral lys− strain; ECM21, extracellular mutant gene 21; Evo, an evolved lys− strain; GSH, reduced glutathione; HPLC, High-Performance Liquid Chromatography; lys−, lysine-requiring mutant; T2, doubling time. (TIF) [file pbio.3000757.s007.tif]

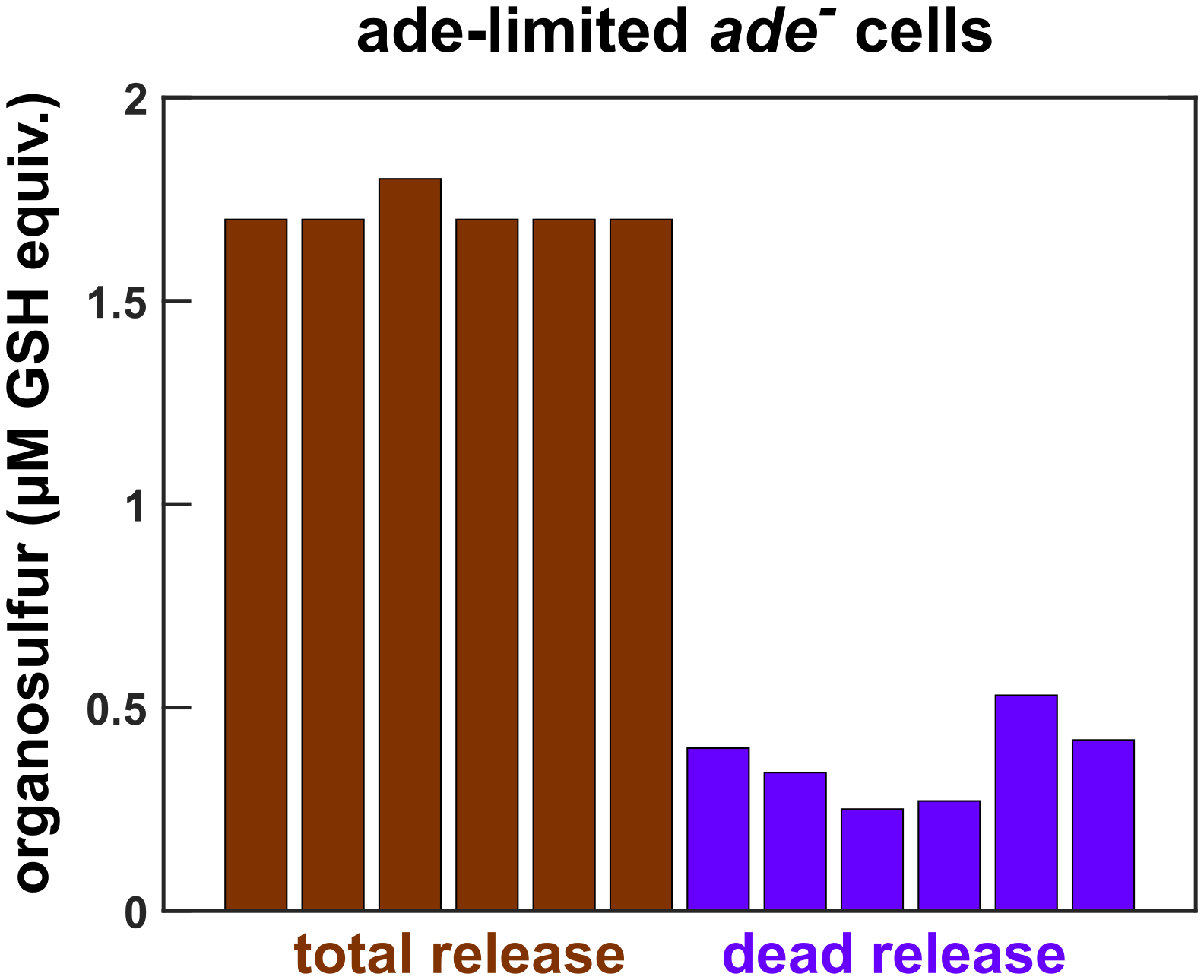

Supplement: S8 Fig — ade− cells (WY1340; WY1598) were grown in adenine-limited chemostats at 8-hour doubling time. After cultures had reached steady-state cell density (approximately 71–73 hours), a sample was taken to assay for total cell density, live-cell density, and dead-cell density using flow cytometry. Simultaneously, another sample was filtered to harvest supernatant, and a third sample was taken to harvest cells to make extracts. Total organosulfurs in supernatants (brown) and cell extracts were measured via the met10-based bioassay. From total organosulfur in extracts and the total number of cells harvested, we calculated fmole organosulfurs per cell. We inferred the contribution to total release by dead cells by multiplying dead-cell density with fmole organosulfur/cell. Six independent experiments are plotted (S5 Data). ade−, adenine-requiring mutant; GSH equiv., reduced glutathione equivalent; met−, methionine-requiring mutant. (TIF) [file pbio.3000757.s008.tif]

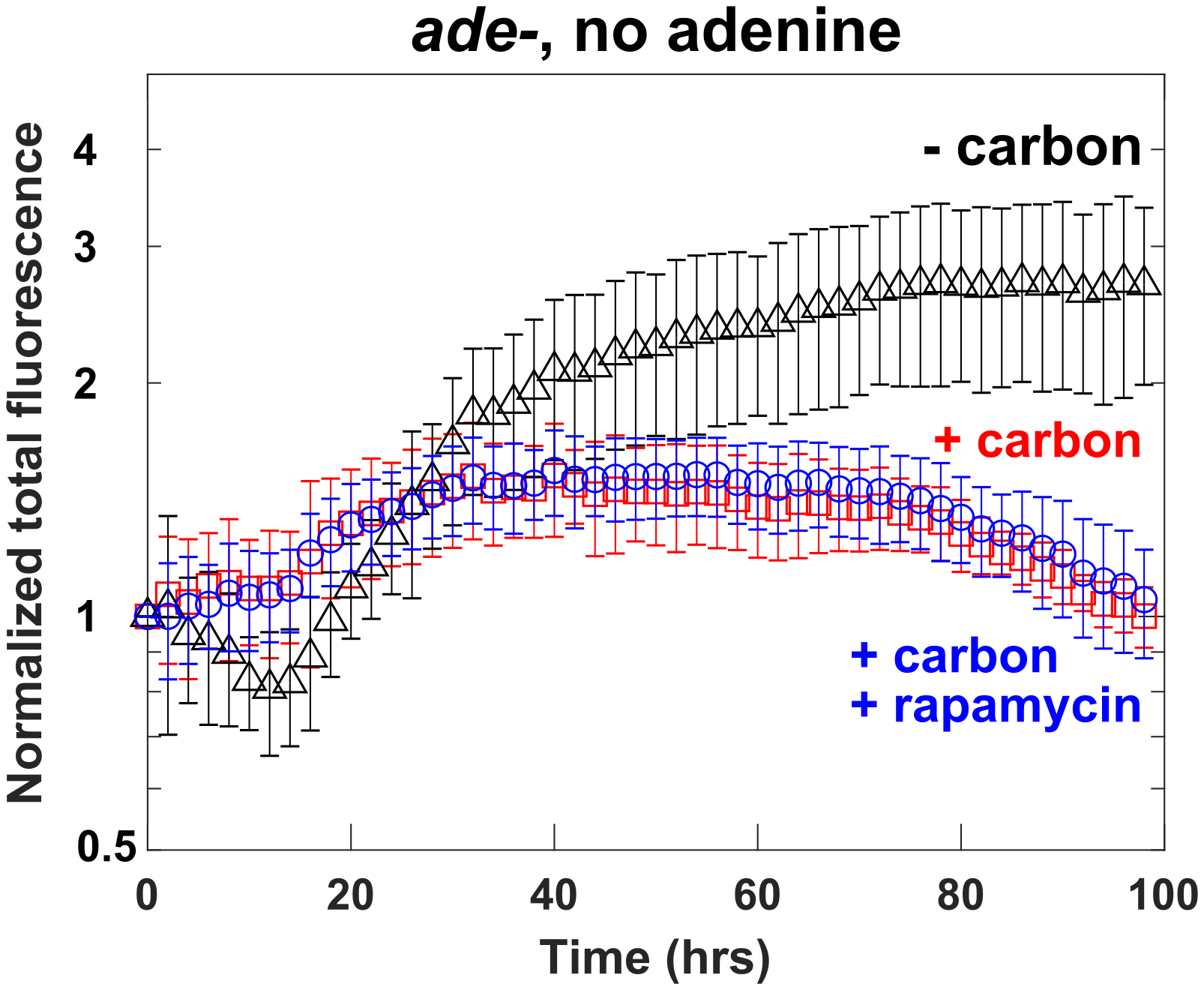

Supplement: S9 Fig — Multiple lines of evidence suggest that adenine-limited ade− cells suffer nutrient–growth dysregulation. In our previous work, we found that in very low concentrations (approximately 0.1–0.2 μM) of hypoxanthine (which can be converted to adenine by cells), ade− cells initially divided but then died at a faster rate than during adenine starvation, consistent with nutrient–growth dysregulation during adenine limitation (Fig 3C in [27]). Here, exponentially growing ade− cells (WY1340) were washed in sterile water and cultured in minimal medium lacking adenine, either with glucose (“+ carbon,” red) or without glucose (“− carbon,” black) for 24 hours before imaging in the same medium. For the rapamycin sample (“+ carbon + rapamycin,” blue), rapamycin was added at the beginning of imaging. After an initial long lag, ade− cells died (red). Similar to lys− cells, cell death during adenine starvation was mitigated by removing glucose (compare red with black). Unlike lys− cells, cell death was not inhibited by rapamycin (compare red with blue). This suggests that during purine starvation, although TORC1 is already inactivated, other pathways (such as Ras/PKA) are still active, contributing to nutrient–growth dysregulation. Error bars correspond to 2 standard deviations from 6 replicate wells. All data are available in S15 Data. ade−, adenine-requiring mutant; lys−, lysine-requiring mutant; PKA, protein kinase A; TORC1, target of rapamycin 1 (TIF) [file pbio.3000757.s009.tif]

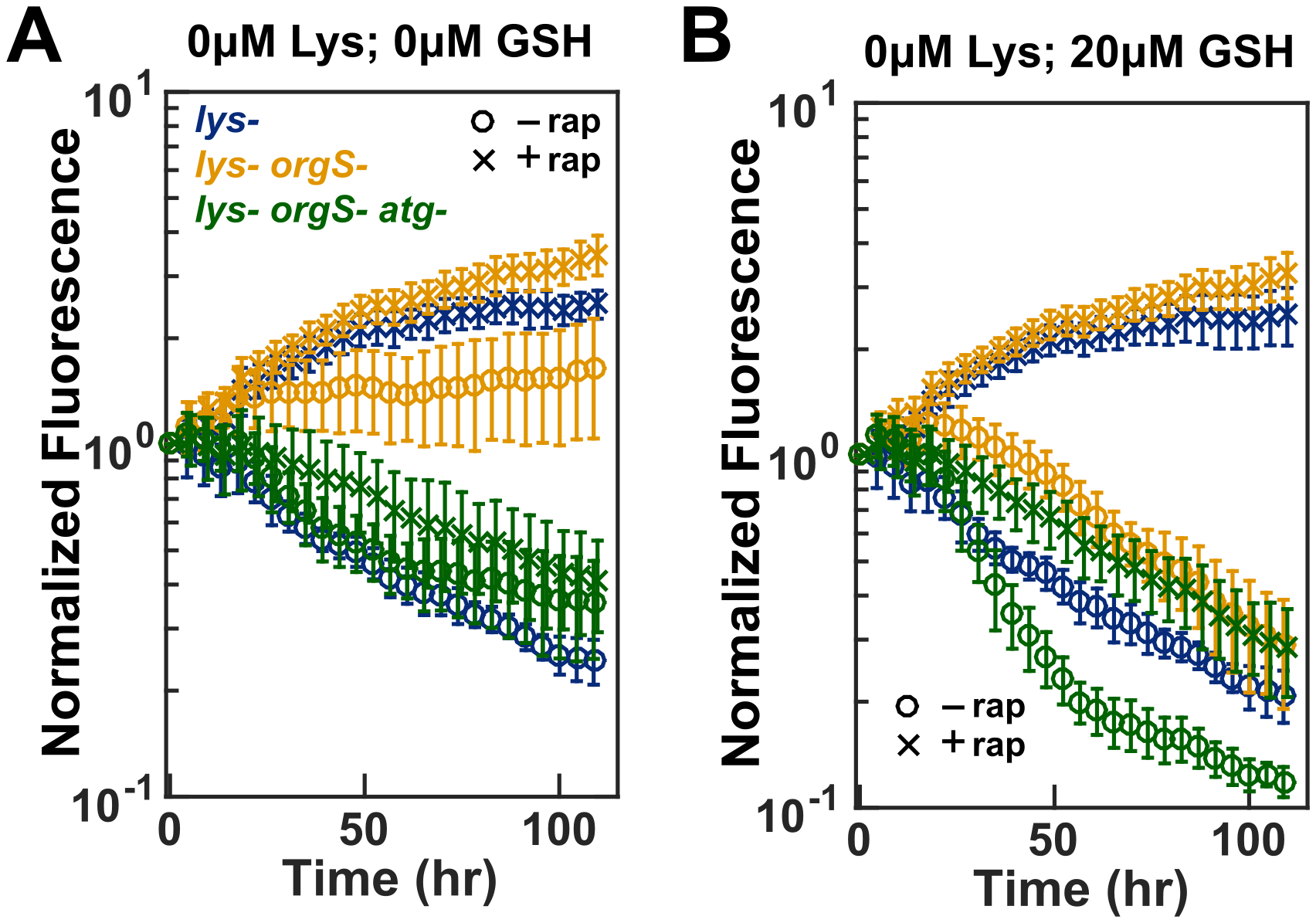

Supplement: S10 Fig — lys− (WY2429, blue), lys−orgS− (WY1604, orange), and lys−orgS−atg5− (WY2370, green) were grown to exponential phase in SD supplemented with excess lysine (164 μM) and excess GSH (134 μM). These cells were washed and starved in SD without lysine or GSH for 5 hours prior to imaging in indicated conditions with 1 μM rapamycin (crosses) or without rapamycin (circles). Total fluorescence normalized against time zero are plotted. All plotted data can be found in S8 Data. (A) When GSH was limited, lys−orgS− survived better than lys− (orange circles above blue circles). TORC1 inhibition by rapamycin improved the survival of both lys−orgS− and lys− to comparable levels (orange and blue crosses). lys−orgS−atg5− survived poorly even when TORC1 was shut down (green crosses). (B) At high GSH, both lys−orgS− and lys− survived poorly (orange circles trending down at a similar slope as blue circles), and this poor viability was rescued by rapamycin (blue and orange crosses). lys−orgS−atg5− survived poorly in the presence or absence of rapamycin (green). The approximately 2-fold initial increase in the top curves was due to cell swelling [27]. Error bars represent 2 standard deviations from 4 positions in a well. atg5, autophagy-related gene 5 mutant; GSH, reduced glutathione; lys−, lysine-requiring mutant; rap, rapamycin; orgS−, organosulfur-requiring mutant; SD, synthetic minimal glucose medium; TORC1, target of rapamycin complex 1 (TIF) [file pbio.3000757.s010.tif]

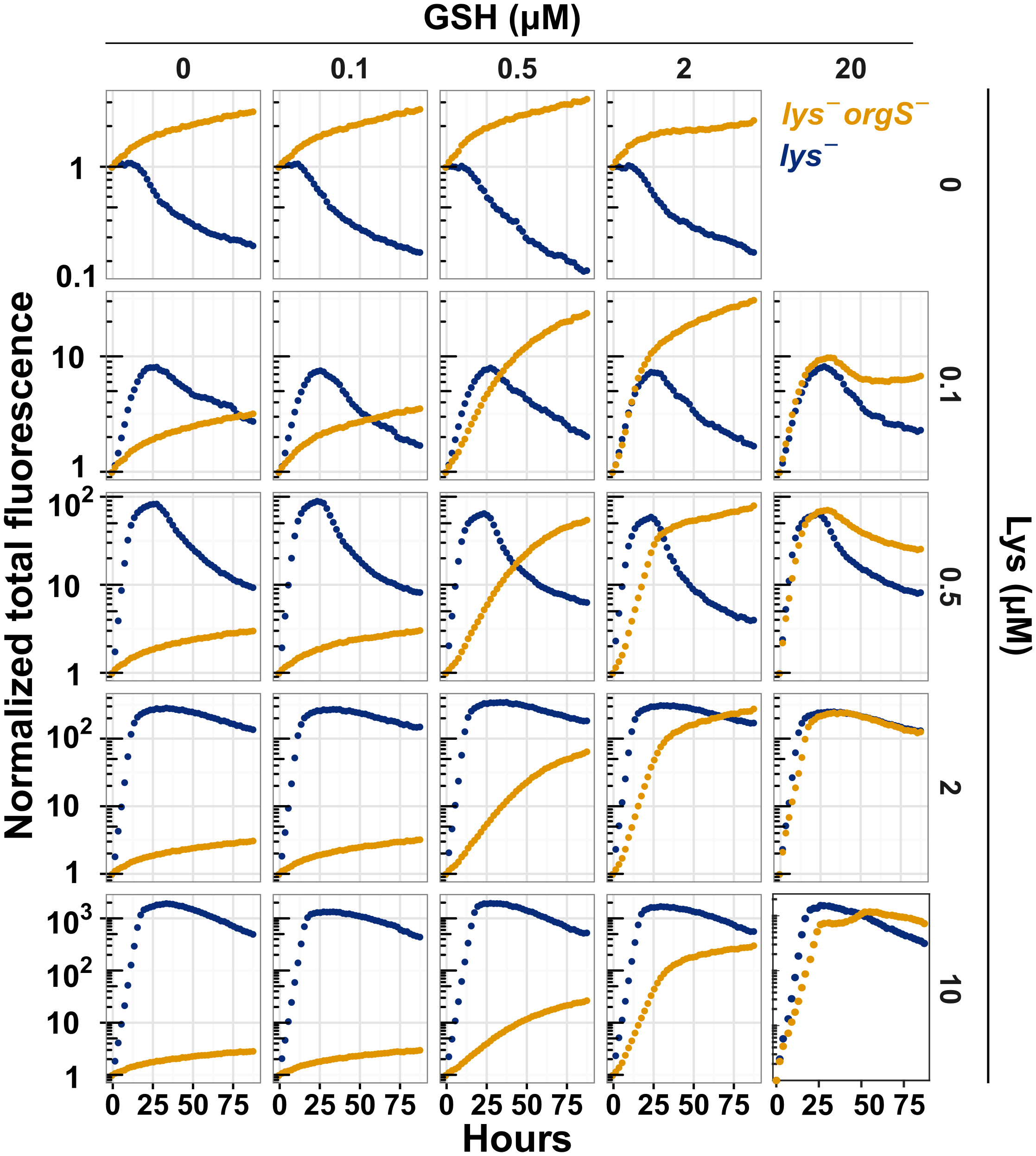

Supplement: S11 Fig — lys− (WY2429, blue) and lys−orgS− (WY1604, orange) cells were grown in SD + excess lysine (164 μM) and excess GSH (134 μM) to exponential phase. These cells were washed and starved in SD for 24 hours and imaged in various concentrations of GSH and lysine. During the growth phase, lys− grew faster than lys−orgS− in most cases. After lysine was exhausted, lys−orgS− survived better than lys−. Note that in the microscopy assay, the minimal medium did not contain GSX or other excreted compounds found in culture supernatants, and thus, results are not directly comparable to competition experiments. Regardless, and consistent with the competition experiment, lys−orgS− cells grew faster than lys− cells under certain conditions. In this experiment, at 2 μM GSH and 0.1 μM lysine, the maximal growth rate achieved by lys−orgS− was 0.153 ± 0.009/hour (mean ± 2 standard error of mean), greater than the 0.133 ± 0.004/hour achieved by lys−. Fluorescence intensities of various time points are normalized against that at time zero. Plotted data can be found in S17 Data. GSH, reduced glutathione; GSX, glutathione-S-conjugate; lys−, lysine-requiring mutant; orgS−, organosulfur-requiring mutant; SD, synthetic minimal glucose medium. (TIF) [file pbio.3000757.s011.tif]

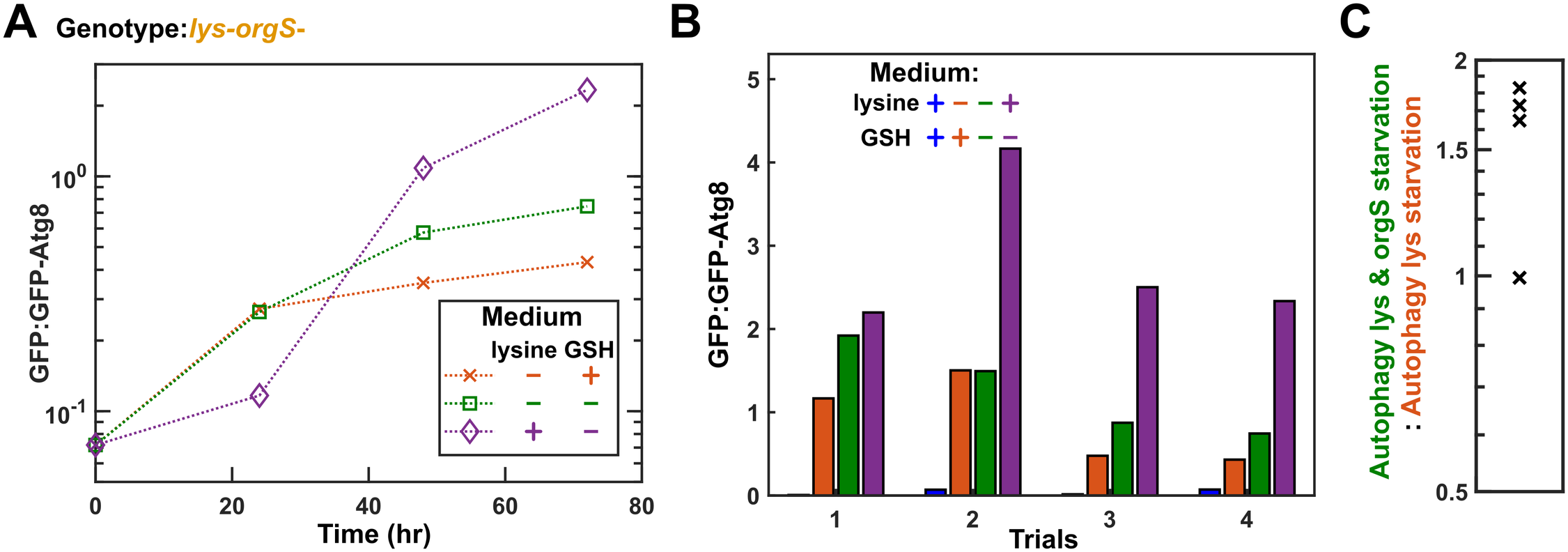

Supplement: S12 Fig — The ideal comparison of autophagy between lys− and lys−orgS− cells has multiple technical difficulties. Cells should ideally be cultured in an environment that mimics the original evolutionary environment (i.e., in low concentrations of lysine and organosulfurs). This means that lys−orgS− cells would need to be cultured in a chemostat dual-limited for sulfur and lysine. However, the theory of chemostat is based on single-nutrient limitation [35], and ensuring dual limitation is nontrivial. Thus, we assayed autophagy in batch starvation cultures. Unfortunately in batch starvation cultures, it can be difficult to compare autophagy activities between different genotypes; e.g., the kinetics of autophagy and death differed drastically between lys− and lys−orgS−. When supplied with excess lysine and no organosulfurs, lys−orgS− cells continued to divide for multiple rounds using internal organosulfur storage, and autophagy induction was very slow. In contrast, lysine-starved lys− cells died quickly. Thus, a comparison between the 2 genotypes is difficult. For these reasons, we compared autophagy activities in batch cultures of a single genotype (lys−orgS−; WY2520) under different starvation conditions. Using the GFP-Atg8 cleavage assay, we observed a moderate increase in autophagy when lys−orgS− cells were starved for both lysine and organosulfurs as opposed to only lysine starvation. Plotted data are available in S16 Data. (A) Time course of autophagy induction during lysine starvation (orange), organosulfur starvation (purple), and dual starvation (green). (B) Results from 4 trials (the fourth trial is identical to A). Autophagy of exponential cultures (first bar in each set) and of singly or doubly starved cultures at 72 h (second to fourth bars in each set) are plotted. (C) Autophagy activity was generally higher in cells starved for both lysine and organosulfurs than in cells starved for lysine only. A one-sample t test against the null hypothesis of identical autopha [file pbio.3000757.s012.tif]

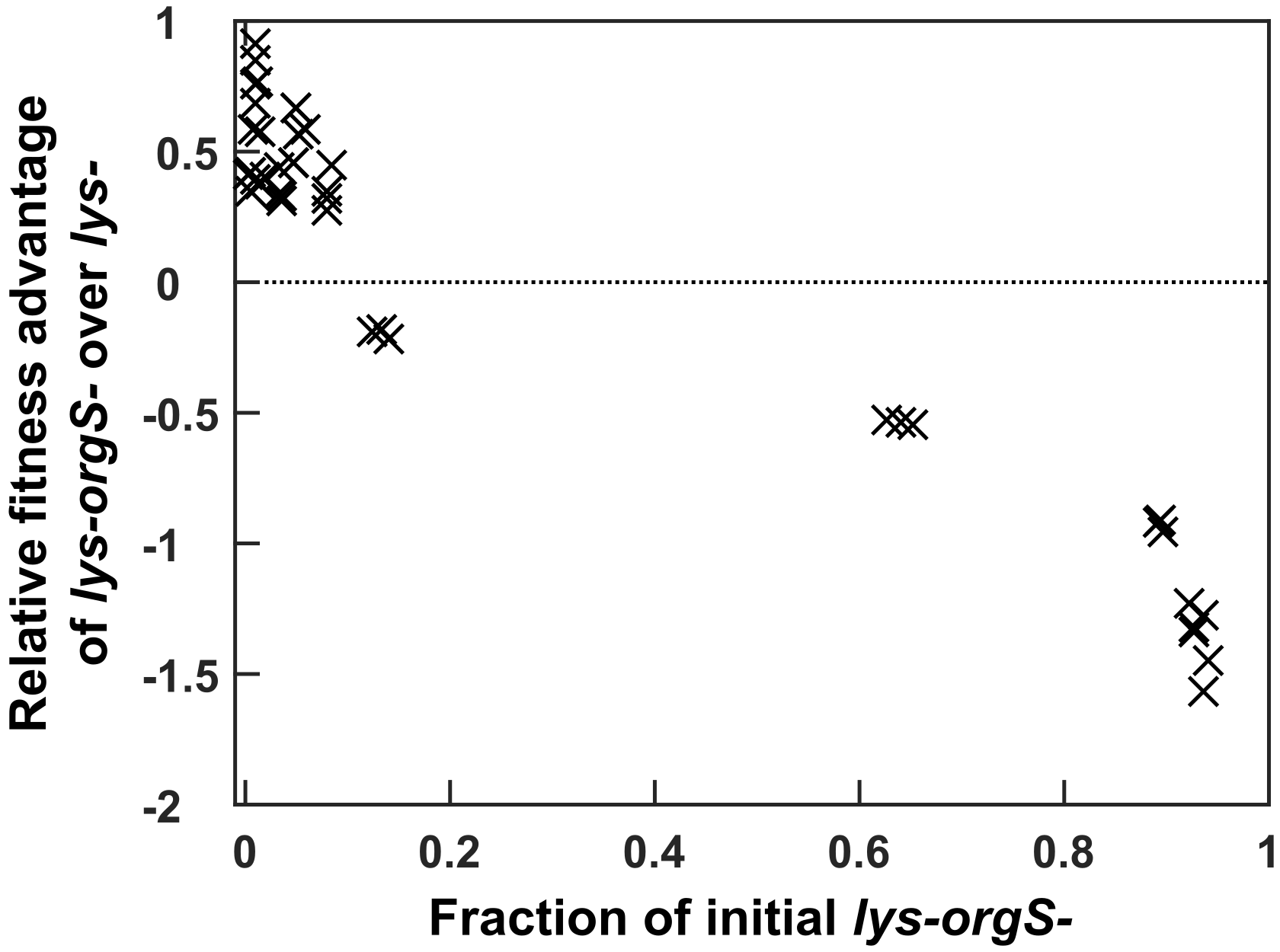

Supplement: S13 Fig — BFP-tagged lys−orgS− (WY2072 or WY2073) and mCherry-tagged lys− (WY2039 or WY2045) were placed in competition in a lysine-limited environment by coculturing with a lysine-releasing strain (WY1340). Strain ratios over time were measured by flow cytometry (Fig 6C). For each trajectory, we computed the slope of ln(lys−orgS−/lys−) over 3 consecutive time points and chose the steepest slope. Since our time unit was generation, we divided this slope (/generation) by ln2/generation and obtained a dimensionless number representing the relative fitness difference between lys−orgS− and lys−. We then plotted the relative fitness difference against the fraction of lys−orgS− at the beginning of the time window used to calculate the steepest slope. Dotted line marks equal fitness between the 2 strains. The fitness advantage of lys−orgS− over lys− decreases as the fraction of lys−orgS− increases (i.e., negative-frequency–dependent). Plotted data are provided in S9 Data. BFP, blue fluorescent protein; lys−, lysine-requiring mutant; orgS−, organosulfur-requiring mutant. (TIF) [file pbio.3000757.s013.tif]

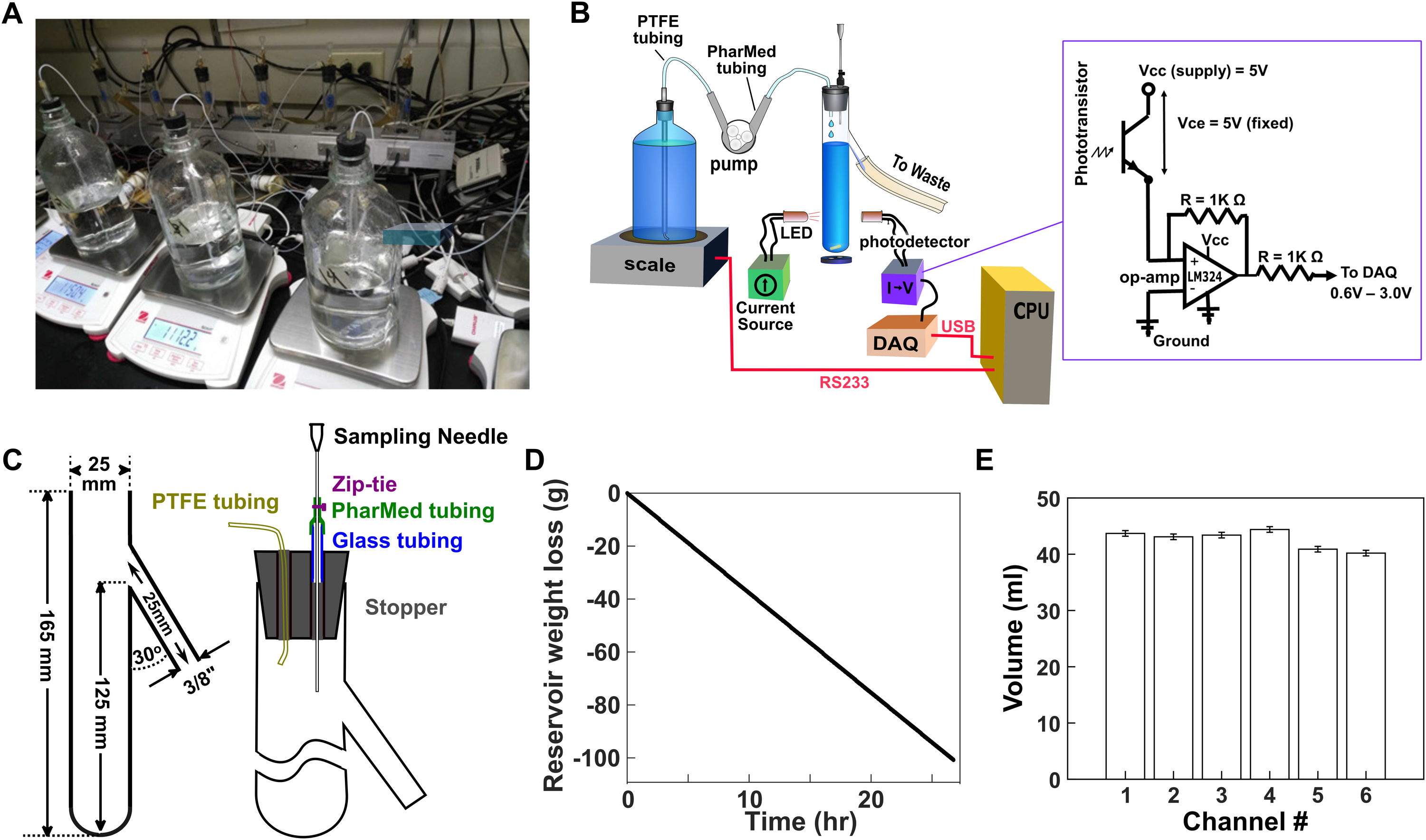

Supplement: S14 Fig — The continuous culturing device (A) consists of 6 channels that can independently operate as a chemostat or a turbidostat. Each channel (B) consists of a culturing vessel (center), a magnetic stirrer, an LED-phototransistor optical detector for OD measurement, a computer-activated pump, a media reservoir (left), and a scale for measuring media reservoir (and flow rate). A LabView program running on the CPU uses data from the scale or the optical detector to control the pump, which maintains a constant average OD in the turbidostat mode, or a constant average flow rate in the chemostat mode. Each vessel (C) consists of a Pyrex test tube modified by adding a waste outlet of adequate diameter and slope to ensure a reliable flow of waste driven by gravity. The vessel’s rubber stopper had a sampling port consisting of a needle that can be raised and lowered through a segment of PharMed tubing, which was held in place by glass tubing inserted into the stopper. The tightness of the seal between the sampling needle and PharMed tubing can be adjusted using a zip-tie, allowing easy motion of the needle while maintaining its position when stationary. The 6 vessels, stirrers, and photodetectors are held in position in a frame cut from an aluminum bar. The signal from each phototransistor was converted to a voltage using an op-amp current to voltage converter (box in B). (D) Constant flow rates in chemostats. An example is shown. (E) Culturing vessel volume (depth approximately 125 mm) averages 43 ml. Individual culturing vessel volume was used for converting doubling time to flow rate and was measured to approximately 0.5-ml resolution (limited by minimum outflow drop size; error bar). Data for D–E can be found in S18 Data. CPU, Central Processing Unit; DAQ, Data Acquisition system; LED, light-emitting diode; OD, optical density; PTFE, Polytetrafluoroethylene. (TIF) [file pbio.3000757.s014.tif]
