# Supplementary material for: Metabolic excretion associated with nutrient–growth dysregulation promotes the rapid evolution of an overt metabolic defect
Source: PLoS Biol. 2020 Aug 24;18(8):e3000757. doi: 10.1371/journal.pbio.3000757 (PMC7470746; doi:10.1371/journal.pbio.3000757)
Supplement: S1 Text — We discuss how our model of nutrient–growth regulation (Fig 1) may be reconciled with seemingly conflicting biochemistry studies on the response of TORC1 to unnatural limitation [83,84]. TORC1, target of rapamycin complex 1. (DOCX) [file pbio.3000757.s015.docx]

## S1 Text. Additional perspectives on nutrient-growth regulation.

Although some biochemistry results seemed to conflict with the nutrient-growth regulation model (Fig 1), we can offer alternative explanations for these results. For example, during unnatural limitation of leucine, histidine, or lysine, TORC1 seemed to be inactivated toward the one or two tested substrates [85,86]. We argue that TORC1 could be active against other substrates. Consistent with this notion, inactivating TORC1 via rapamycin rescued poor viability of *lys^-^* cells during lysine starvation (Fig 2A). In addition, the Ras/PKA pathway could also be active, contributing to nutrient-growth dysregulation. As another example, leucine-sensing mechanisms have been found in yeast [19,20], suggesting that leucine limitation may be interpreted as a natural limitation. Regardless of the leucine-sensing mechanism, *leu^-^* cells still suffered poor viability during leucine starvation compared to during natural starvation [6,23,24]. One possibility is that growth inhibition by leucine starvation is overwhelmed by growth promotion by natural nutrients.

We argue that when different assays generate conflicting results, fitness phenotypes (e.g. cell growth or death rate) should always be given the highest weight. This is because 1) fitness is the final read-out of all the known and the unknown biochemistry inside and outside of a cell, and 2) natural selection acts on fitness.
