# Supplementary material for: Metabolic excretion associated with nutrient–growth dysregulation promotes the rapid evolution of an overt metabolic defect
Source: PLoS Biol. 2020 Aug 24;18(8):e3000757. doi: 10.1371/journal.pbio.3000757 (PMC7470746; doi:10.1371/journal.pbio.3000757)
Supplement: S1 Raw Images — (PDF) [file pbio.3000757.s036.pdf]

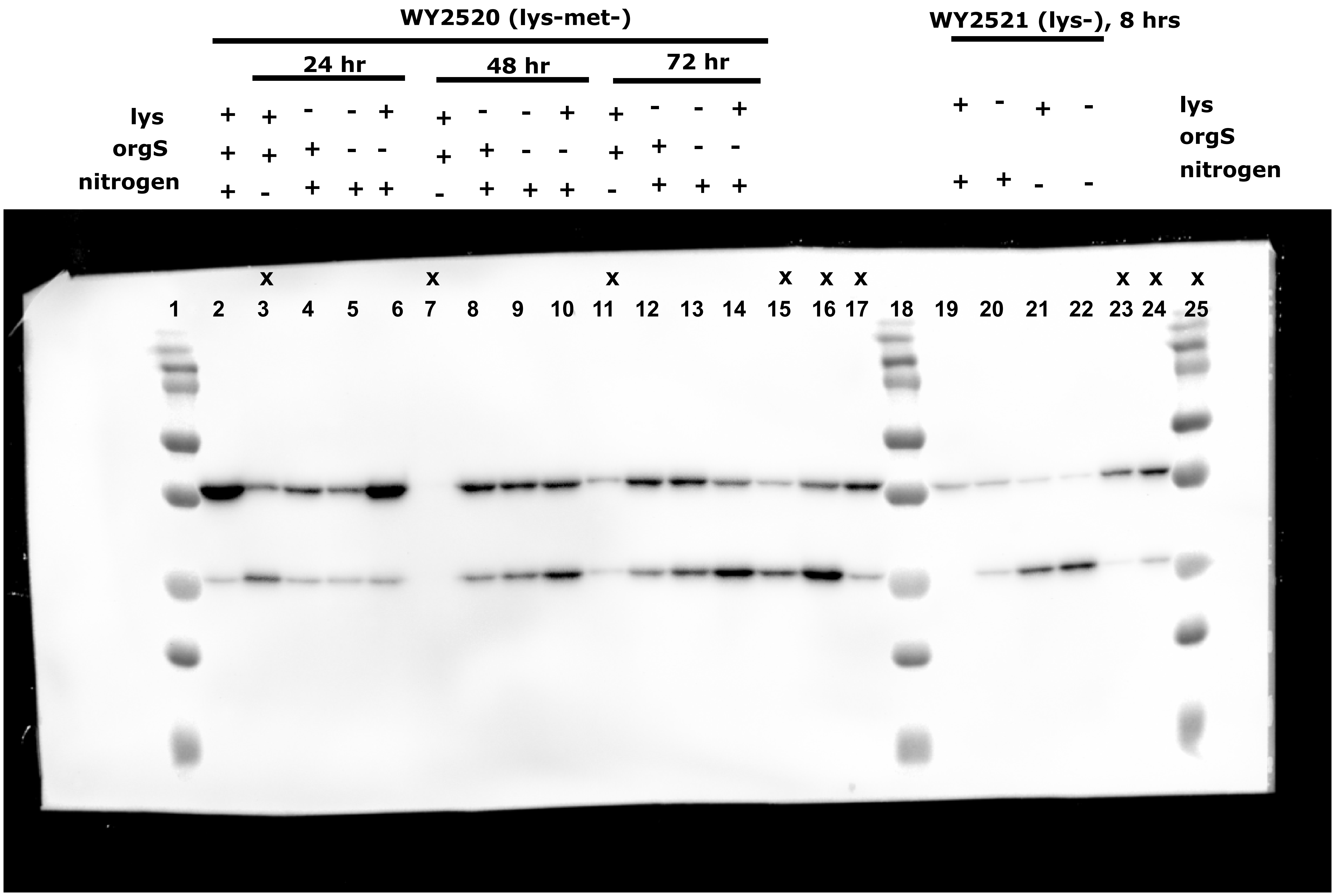

Lanes 18-22 from this blot were used in Fig 2B
18: Molecular weight ladder
19: exponential WY2521 before starvation
20: WY2521 starved only for lysine for 8 hours
21: WY2521 starved only for nitrogen for 8 hours
22: WY2521 starved for both lysine and nitrogen for 8 hours

Band intensities from lanes 1-14 excluding those marked with an "x" were used for anaysis plotted in S12A Fig and provided in S16 Data
1,18: Molecular weight ladder
2: exponential WY2520 before starvation
4,8,12: WY2520 starved of lysine for 24, 48 and 72 hours, respectively
5,9,13: WY2520 starved of both lysine and organosulfurs for 24, 48 and 72 hours, respectively
6,10,14: WY2520 starved only 0f organosulfurs for 24, 48 and 72 hours, respectively

The detailed protocol is provided under "Autophagy Assay" in Methods
TCA-precipitated cell lysates from S. cerevisiae were loaded in each lane
Blots were treated with anti-GFP (JL-8) primary and HRP-conjugated anti-mouse secondary
Image is an overlay of the colorimetric image of the protein ladder and chemiluminescence imaging of the stained protein bands

gel date: 2020-02-26

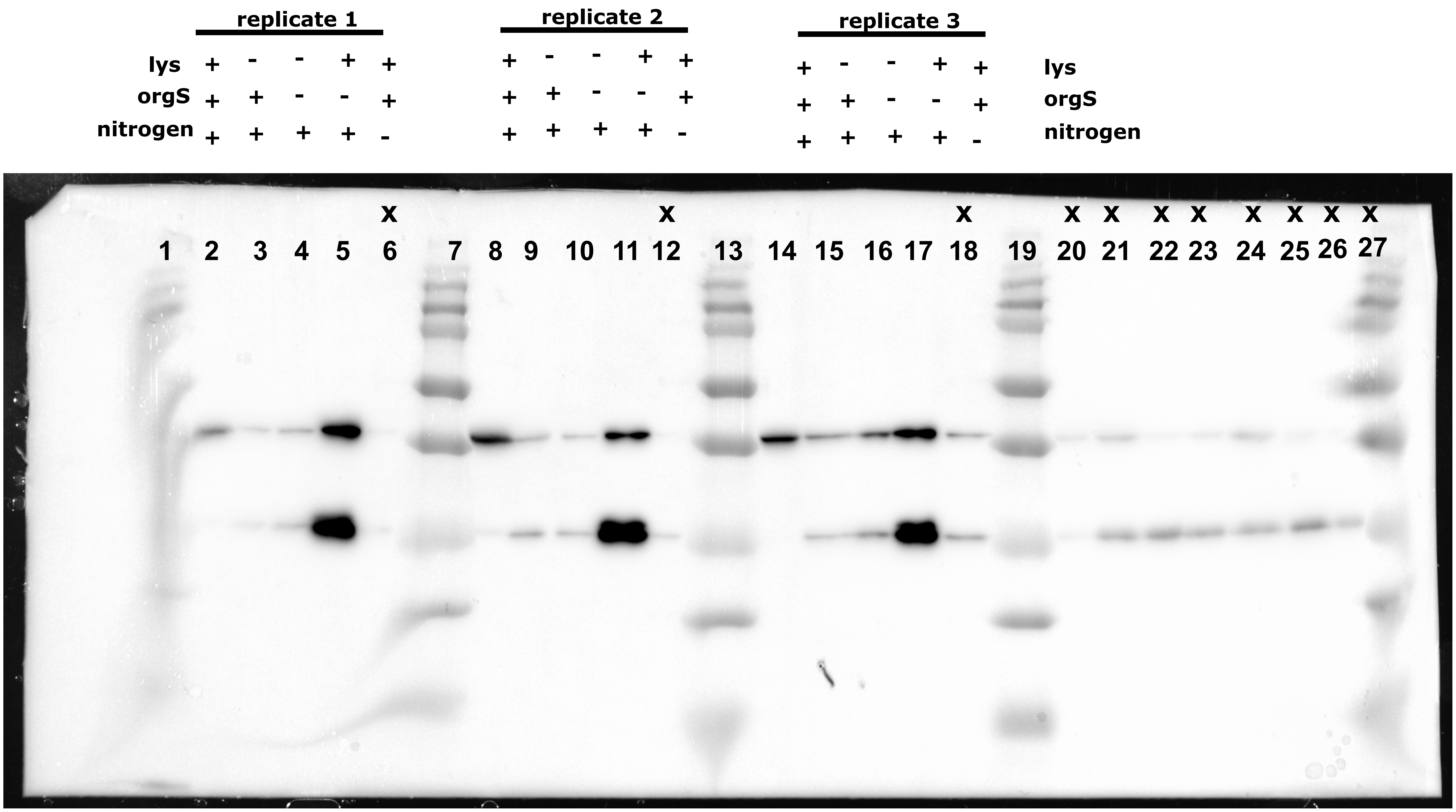

Band intensities from all lanes except those marked with an "x" were used for anaysis plotted in S12B-C Figs and provided in S16 Data.

1,7,13,19: Molecular weight ladder

2,8,14: exponential WY2520 before starvation

3,9,15: WY2520 starved of lysine for 72 hours

4,10,16: WY2520 starved of both lysine and organosulfurs for 72 hours

5,11,17: WY2520 starved only 0f organosulfurs for 72 hours

The detailed protocol is provided under "Autophagy Assay" in Methods

TCA-precipitated cell lysates from S. cerevisiae were loaded in each lane

Blots were treated with anti-GFP (JL-8) primary and HRP-conjugated anti-mouse secondary

Image is an overlay of the colorimetric image of the protein ladder and chemiluminescence imaging of the stained protein bands

gel date: 2020-03-03

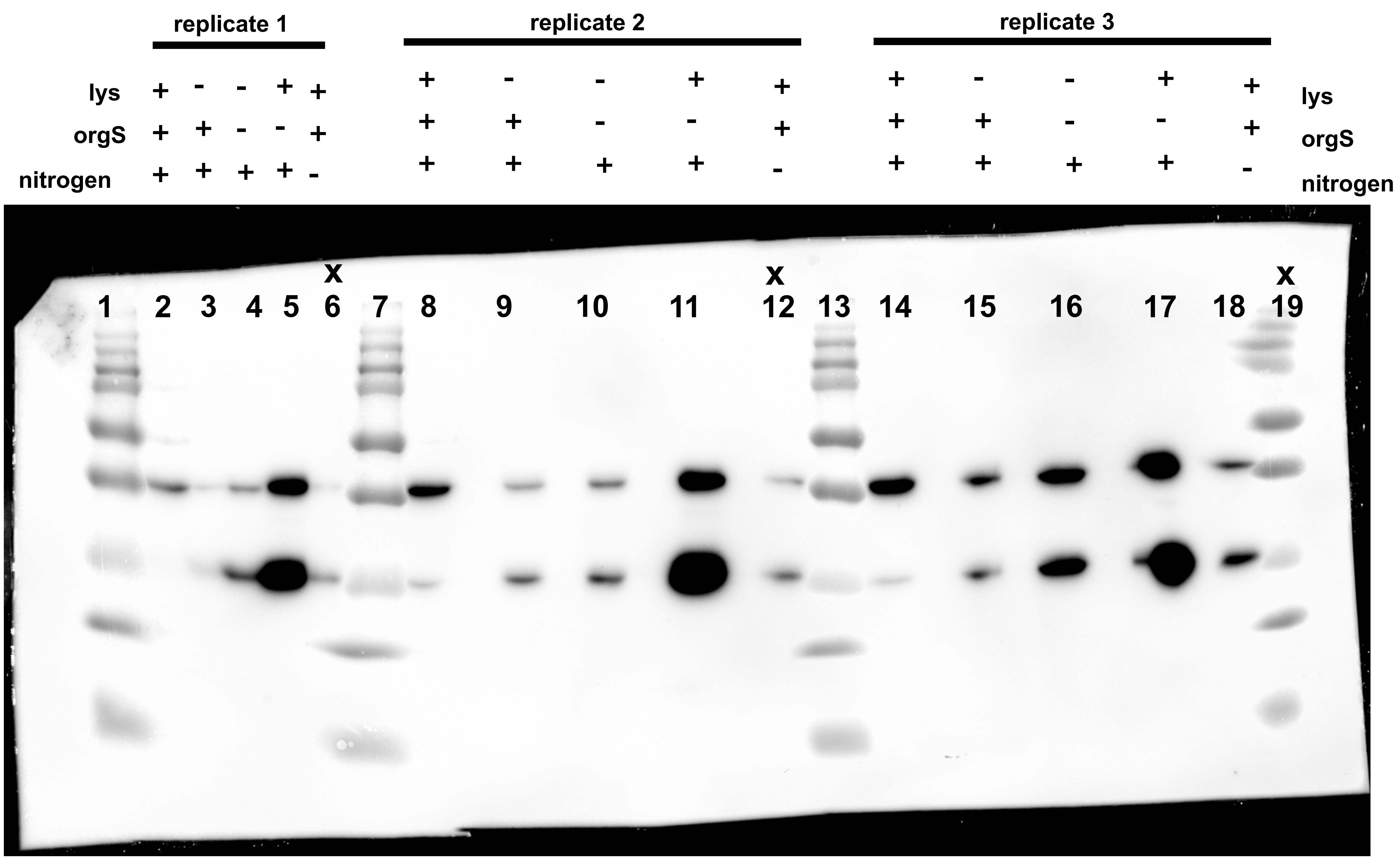

Band intensities from all lanes except those marked with an "x" were used for anaysis plotted in S12B-C Figs and provided in S16 Data.

1,7,13,19: Molecular weight ladder
2,8,14: exponential WY2520 before starvation
3,9,15: WY2520 starved of lysine for 72 hours
4,10,16: WY2520 starved of both lysine and organosulfurs for 72 hours
5,11,17: WY2520 starved only of organosulfurs for 72 hours

The detailed protocol is provided under "Autophagy Assay" in Methods
TCA-precipitated cell lysates from S. cerevisiae were loaded in each lane
Blots were treated with anti-GFP (JL-8) primary and HRP-conjugated anti-mouse secondary
Image is an overlay of the colorimetric image of the protein ladder and chemiluminescence imaging of the stained protein bands
